# Fabrication-conscious neural network based inverse design of single-material variable-index multilayer films

**DOI:** 10.1515/nanoph-2022-0537

**Published:** 2023-01-30

**Authors:** Omer Yesilyurt, Samuel Peana, Vahagn Mkhitaryan, Karthik Pagadala, Vladimir M. Shalaev, Alexander V. Kildishev, Alexandra Boltasseva

**Affiliations:** Elmore Family School of Electrical and Computer Engineering, Birck Nanotechnology Center and Purdue Quantum Science and Engineering Institute, Purdue University, West Lafayette, IN 47907, USA; The Quantum Science Center (QSC), a National Quantum Information Science Research Center of the U.S. Department of Energy (DOE), Oak Ridge, TN 37931, USA

**Keywords:** deep learning, fabrication-in-loop, inverse design, nanophotonics

## Abstract

Multilayer films with continuously varying indices for each layer have attracted great deal of attention due to their superior optical, mechanical, and thermal properties. However, difficulties in fabrication have limited their application and study in scientific literature compared to multilayer films with fixed index layers. In this work we propose a neural network based inverse design technique enabled by a differentiable analytical solver for realistic design and fabrication of single material variable-index multilayer films. This approach generates multilayer films with excellent performance under ideal conditions. We furthermore address the issue of how to translate these ideal designs into practical useful devices which will naturally suffer from growth imperfections. By integrating simulated systematic and random errors just as a deposition tool would into the optimization process, we demonstrated that the same neural network that produced the ideal device can be retrained to produce designs compensating for systematic deposition errors. Furthermore, the proposed approach corrects for systematic errors even in the presence of random fabrication imperfections. The results outlined in this paper provide a practical and experimentally viable approach for the design of single material multilayer film stacks for an extremely wide variety of practical applications with high performance.

## 1 Introduction

Multilayer films are simple, planar structures that have found a remarkably wide application space in optics including photovoltaics [1], 1-D photonic crystals [2], anti-reflection coatings [3], optical filters [4], and mirrors [5], to name a few. Traditionally, these structures are made of alternating layers of materials with different optical properties. This limits the design space by only allowing for a limited number of refractive indices for each layer. An alternative approach is to use materials and growth techniques that allow continuous and gradual changes in refractive index. This allows for the growth of multilayer films where each layer is made of the same material, but the index is varied by growth conditions. This approach has several major demonstrated advantages, the first is that the films produced are monolithic, leading to improved mechanical performance particularly enhanced strength and toughness [6]. Secondly, films produced this way have no internal interfaces leading to a reduction of light scattering and the elimination of side-band harmonics [7, 8]. Finally, such films are made of a single material, which can greatly simplify any further processing such as patterning. A variety of material platforms and film growth techniques have been explored for their ability to achieve continuously variable indices of refraction. Porous silicon obtained via electrochemical etching has been extensively used to create inhomogeneous optical filters [9, 10]. However, their application range is limited due to the absorptive nature of silicon at visible wavelengths. Rugate filters have been demonstrated with reactive magnetron sputtering by controlling the oxygen and nitrogen stoichiometry of deposited SixOyNz films [11]. Another common conformal growth technique is chemical vapor deposition. This film growth technique, as its name implies, is the result of the chemical reaction between precursor gasses assisted by heat and in the case of plasma enhanced tools: microwave power. By adjusting the reaction chemistry and growth conditions during CVD deposition, broad refractive index control can be achieved as the resulting films will have different composition stoichiometry. Single-material monolithic bandpass filters made with plasma-enhanced CVD (PE-CVD) silicon nitride were demonstrated by varying the input microwave power [12]. Similarly, rugate filters were grown with PE-CVD using silicon carbon-oxynitride coatings by continuously adjusting N2 and O2 flow into the reaction chamber [13]. Despite numerous successful demonstrations and the unique advantages of optical devices made of a single material, the related scientific publications are limited compared to their multi-material counterparts. One reason for this is that this fabrication approach requires precise and dynamic control of the deposition conditions to achieve the required optical parameters [14].

One of the challenges facing the fabrication of such multilayer film devices are random fabrication imperfections. Algorithms for generating multilayer films robust to random fabrication-related perturbation have been thoroughly investigated. Usually, modification of the algorithm is done at the level of the loss function. Where in addition to basic merit functions such as target transmittance/reflectance spectra, the change due to imperfections in the multilayer film parameters (refractive index and layer thickness) of the merit function is added to the loss term [15, 16]. These terms are scaled and added with the basic figure of merit in the loss function to realize robust and high-performance devices. Similarly, second-order derivatives of the basic figure of merit (sensitivities) of the errors in the layer parameters have been used to generate robust multilayer films [17–19]. More recently, training of auto encoder-based inverse design architectures with imperfections in the EM response of core–shell particles has been investigated for robust design [20]. However, robustness schemes have only proven to be effective for small imperfections in the layer parameters, less than a one percent shift from the ideal structure in the context of multilayer structures. Deposition error compensation has been proposed to address the major imperfections introduced during the deposition of layers [14]. In this method, a layer of the multilayer stack is deposited. Then the refractive index and thickness of the deposited layer is measured. With this information, a new stack is optimized with the measured grown layers parameters fixed. However, for each layer grown, number of layers left for compensation of errors decreases. Thus, towards the end of the multilayer growth, this method of error compensation becomes less effective and places limits on the overall performance that can be achieved. Furthermore, the grown layers will be imperfect themselves preventing full compensation of the errors made in previous layers.

This paper presents a different approach for high-performance and fabrication conscious multilayer film design. We will use a similar method to those proposed in [21–23] where neural network parameters are updated with gradients extracted from an analytical solver, to optimize a multilayer optical stack with a continuously changing refractive index. This method has the advantage of requiring no prior dataset and demonstrates rapid convergence across a variety of target spectra. Additionally, even though an ideal design is found using this method, when it is fabricated, the systematic and random errors introduced during the growth of the monolithic structure will degrade the performance. To address us our method has a second stage of training for our neural network, by using the data from experimentally grown film, the same neural network optimized in the first stage is then trained using experimental data in each training cycle. The result of this second-stage fabrication-in-the-loop training is that the neural network from stage one is further trained to compensate for systematic errors. In this work we demonstrate the flexibility and applicability of this approach across a variety of error types.

## 2 Multilayer optical film optimization

**Figure 1:**
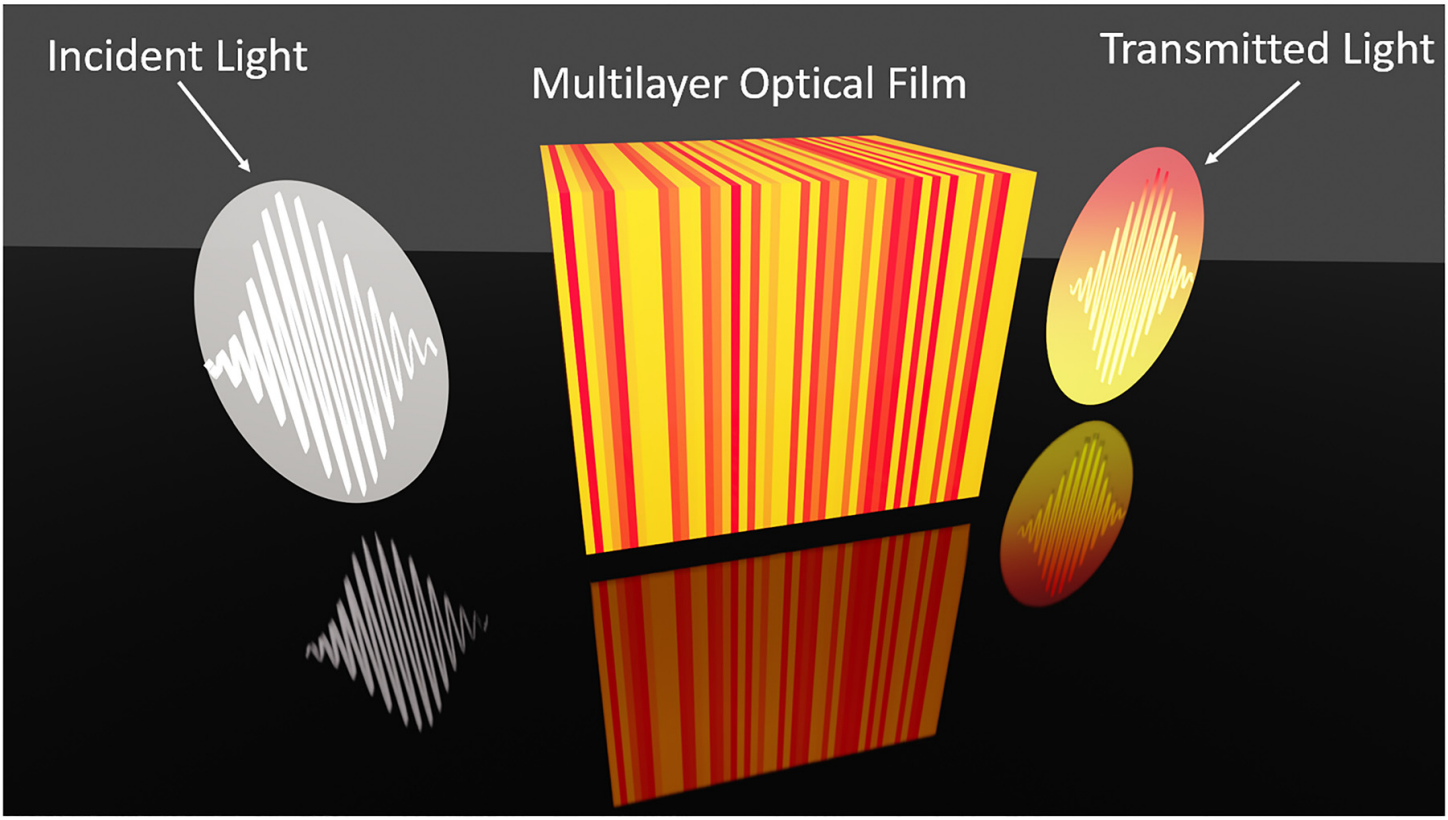
Transmission of unpolarized white light through an optical multilayer film stack. The different colors in the film stack represent the different refractive indices of the different layers.

Design and optimization of multilayer optical films have been of great interest to the scientific community for the past century. The functionality of the devices, enabled by the fast analytical methods to predict their optical response, makes these structures a prime candidate for optimization research. Without loss of generality, the target of this problem is to achieve a given target spectra (transmission/reflection) with a multilayer film of alternating material layers, as shown in Figure 1. Many approaches have been proposed to solve this optimization problem. One such approach is the Fourier method. The refractive-index profile is derived for an inhomogeneous layer of infinite extent having the desired electromagnetic response and then approximated by a finite system of discrete homogeneous layers [24]. Another approach is the needle method [25] which treats the multilayer film as an interference structure whose spectral performance depends on the interference effects among the light waves reflected from its various layer boundaries. Global optimization techniques, such as genetic algorithms [26], simulated annealing [27] etc., have also yielded good results for multilayer film designs. More recently, data-driven approaches such as DL-based methods have been introduced. In our case, the forward problem maps layer parameters (input set) onto the optical response (output set). The uniqueness of the electromagnetic response for a given stack allows for the use of multilayer perceptrons [28] and convolutional neural networks [29]. The inverse design problem treats the target optical response as the input. This is a highly degenerate problem, as many solutions may exist for a given optical response. We refer the reader to Liu et al. for a more detailed analysis of the inverse problem [28]. Since it is impossible to reconstruct multilayer parameters uniquely, optimization techniques are the only effective approach. For approaches employing DL optimization techniques, neural networks are trained to produce multilayer film parameters that exhibit an optical response as close to the desired spectra as possible. Deep reinforcement learning models along with Q-variants have been proposed to optimize the thicknesses of the layers as well as the number of layers [30, 31]. Generative models like conditional variational autoencoders (VAE) have also been applied to anti-reflection grating design. With the addition of active learning to the VAE, generated high-performing designs are then fed back into the dataset to realize minimum grating reflectivity [32]. Most of the methods mentioned up to this point are gradient-based optimizers. Thus, they require the calculation of the local gradients for design parameters adding to the computational complexity. Alternatively, numerical and analytical methods that allow calculation and backpropagation of local gradients are proposed for the efficient optimization of electromagnetic devices. Automatic differentiation and inverse design approaches based on numerical differentiable solvers are shown in [33, 34]. More recently, an analytical solver that enables automatic differentiation for calculating the multilayer film spectra has been shown in [35]. High-performance multilayer optical stacks have been demonstrated by integrating these differentiable solvers into NN-based optimization architectures [23, 35]. Updating the NN parameters with local gradients combines the best of both worlds with high-quality solutions obtained by local gradients and fast convergence enabled by NN [21, 23, 35] Using this central idea, we implemented an NN-based optimizer for multilayer films with continuously changing refractive index. Furthermore, we propose a fabrication-in-loop optimization framework where the fabrication imperfections are integrated into the optimization cycles to enable realistic fabrication of high-performance devices.

## 3 Methods

Deep learning methods for photonics design require a training dataset for the forward and inverse problems typically [36–38]. The computational cost of producing a large training set depends upon the problem’s complexity and therefore, could be prohibitively expensive. Additionally, the direct application of data-driven models does not guarantee the best fit to the target spectrum due to extreme differences between the target spectra and the randomly generated training dataset. To improve performance, the training dataset can include pre-optimized designs. However, this significantly escalates the dataset preparation time since a large set of pre-optimized designs is needed to achieve better performance. Alternatively, NNs can be used for direct optimization without requiring a training dataset. In this configuration, local gradients calculated with a differentiable solver are backpropagated to update NN parameters.

**Figure 2:**
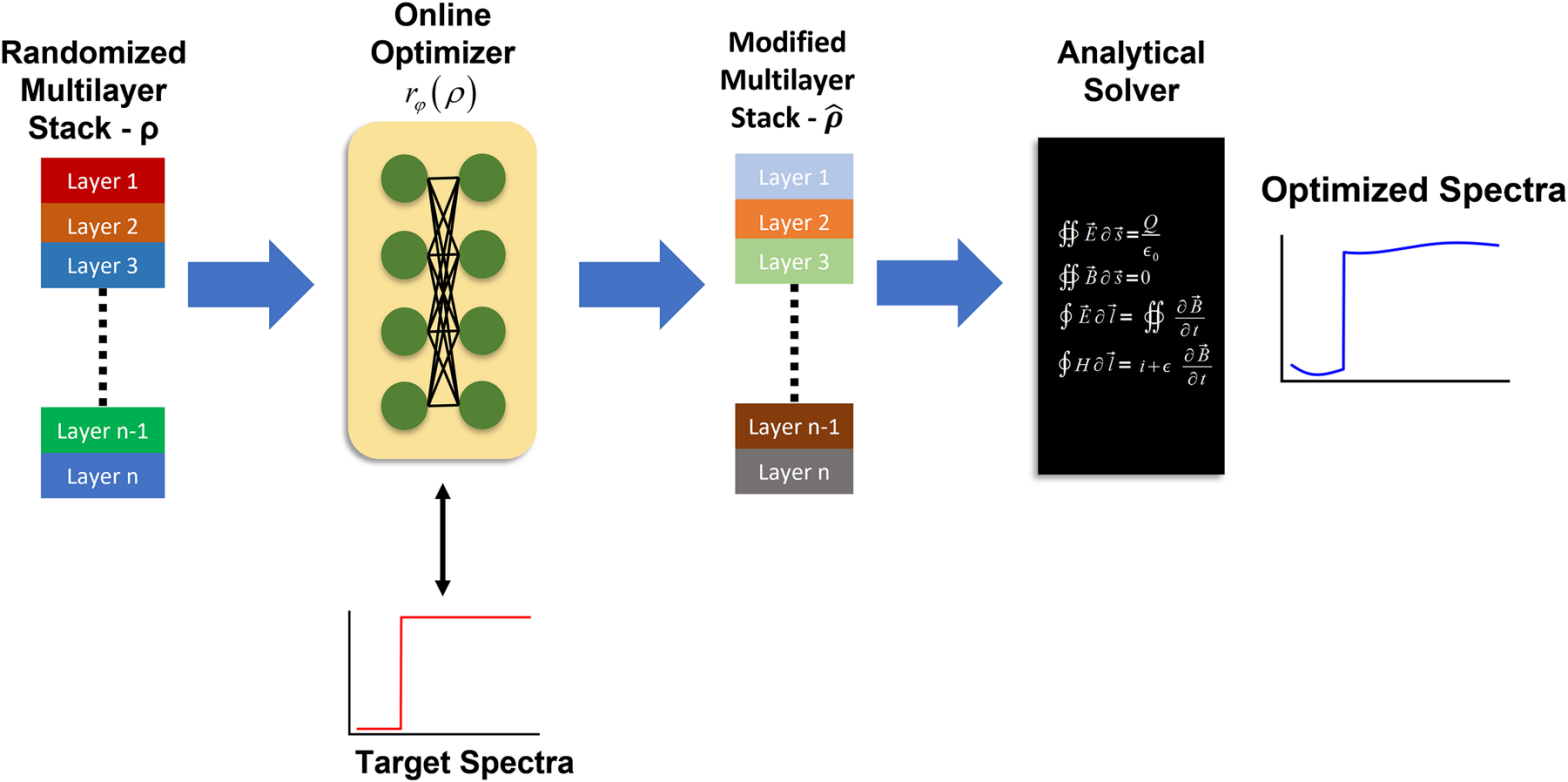
NN based inverse design of multilayer optical films with continuously varying refractive index. An initial randomized set of layer parameters is given to the online optimizer, a fully connected neural network. The online optimizer produces a modified film stack. The optical spectrum of the new film stack is then computed using an analytical transfer matrix method-based solver. The resulting spectra and the target spectra are then used to compute the loss function and the loss is backpropagated to update the online optimizer parameters. This training is done until the online optimizer network is capable of modifying the random input stack into an optimal stack at which point the optimal stack is returned as the optimization solution.

This approach is a NN training method in which a neural network is trained to modify a single input random seed design to produce an optimal design. Our implementation of the NN-based inverse design of multilayer optical films with continuously changing refractive index is shown in Figure 2. The network is trained by feeding in a randomly generated seed design vector (***ρ***) into a fully connected neural network (FCNN) called the online optimizer *r*_*φ*_, with parameter set *φ*. This FCNN produces a modified stack vector $\hat{\rho }$ from the input seed design vector ***ρ***. The optical response of the modified stack (***x***) is then computed using an analytical solver. The error/loss is computed as the mean squared error between the modified stack and target spectra. Finally, the loss is backpropagated through the network to update the biases and weights of the FCNN *φ*. This training sequence is repeated until the neural network is trained to take the input seed design and produce optimal modified design. This is an optimization problem in which the parameters of the FCNN *φ* are updated, given an input seed design ***ρ*** to minimize the mean square error between the output modified stack $\hat{\rho }$ optical response ***x***, and the target response $\hat{x}$ , 

$$\underset{\varphi ,\rho }{\min}\frac{1}{n}\overset{n}{\underset{i=1}{\sum }}{\left(x_{i}-{\hat{x}}_{i}\right)}^{2},\forall \rho_{i}\in \left[\rho_{\min},\rho_{\text{max}}\right]$$

One thing to note is that the seed design ***ρ*** stays the same throughout the optimization. This means that for each training iteration the exact same seed design is fed into the FCNN. As a result, a highly specialized NN is obtained, which produces an optimal design from the given input seed design. Counterintuitively, in this method, the optimization is done on the FCNN, and the optimal design is a byproduct of training the optimal NN.

**Figure 3:**
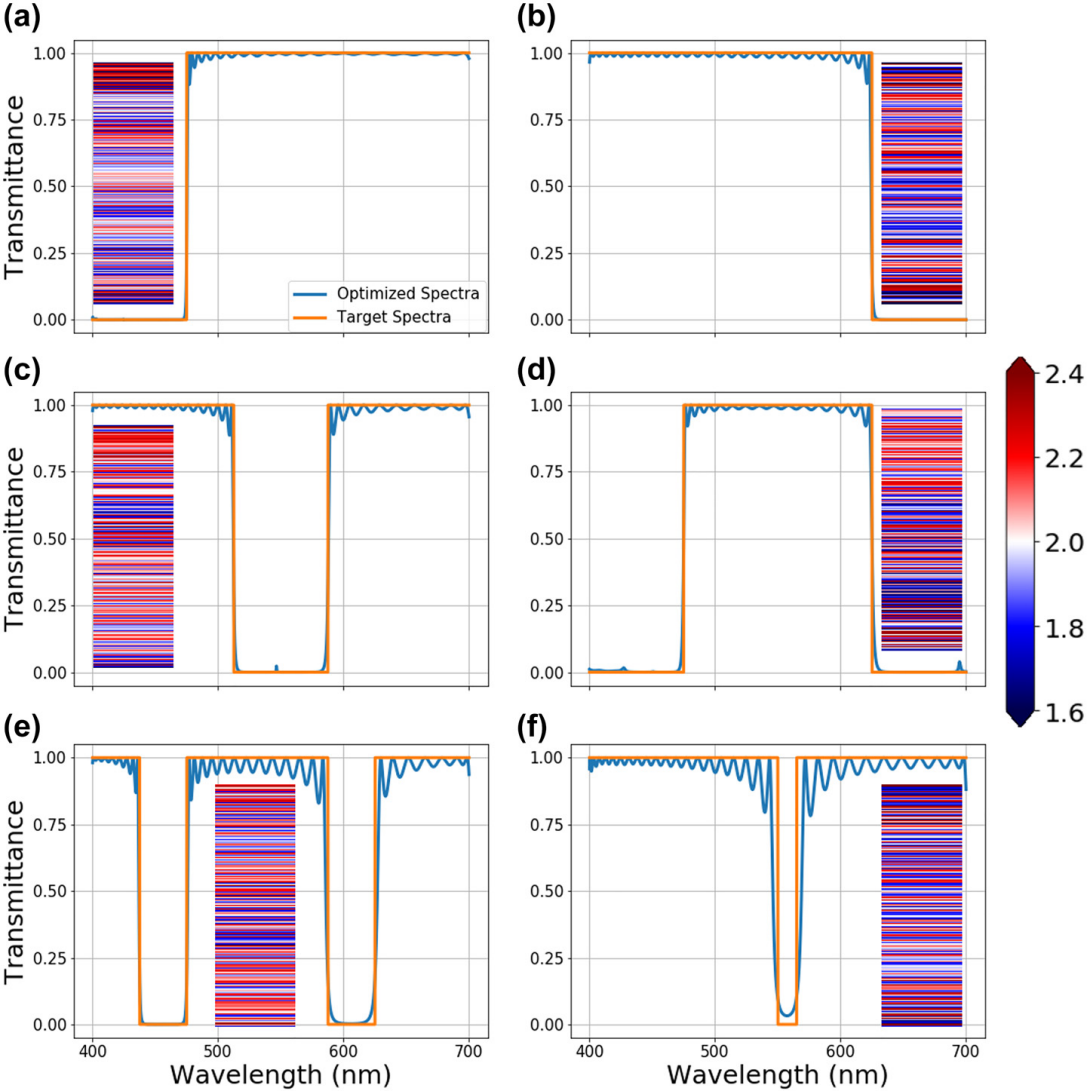
The results of the single material multilayer film optimization with continuously changing refractive index. The generated stacks are optimized for (a) high pass (475 nm cut-off), (b) low pass (625 cut-off), (c) band stop (525–575 nm), (d) band pass (475–625 nm), (e) double-band stop (437.5–475 nm and 587.5–625 nm), (f) and narrow band stop (550–565 nm) optical filters respectively. The insets show the refractive indices of the layers which range from 1.6 to 2.4. The color bar for the range of refractive indices in the stacks is shown on the middle right of the figure. The layer thicknesses and total number of the layers are 30 nm and 200, respectively.

## 4 Results and discussion

### 4.1 Error free optimization

**Figure 4:**
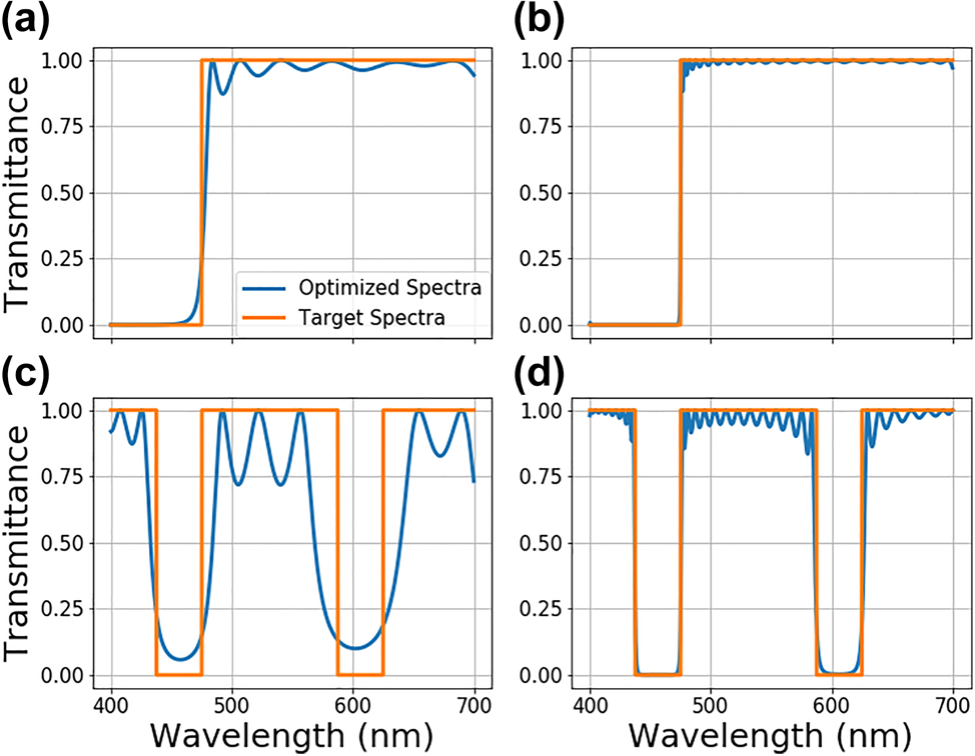
Transmittance spectra comparison between the target spectra (orange) and the generated optimized optical stack spectra (blue) for a long pass filter with a cut-on at 475 nm for (a) 50, and (b) 200-layer structures. The same plots are shown for a dual stop band filter with stop ranges between 437.5 and 475 nm and 587.5–625 nm for (d) 50, and (e) 200-layer structures.

Here, we present NN-based optimization results for single material multilayer films with continuously changing refractive index. In the first study, the layer thicknesses are fixed to 30 nm, while their refractive indices can vary within a user-defined range. Our previous work [39] demonstrated that continuously variable refractive indices are achievable with high-density plasma chemical vapor deposition (HDPCVD) grown SiNx by varying the chamber stoichiometry. Based on this experimentally collected data, we set the refractive index range achievable to be between 1.6 and 2.4. The NN-based optimization starts with an initial input of a random multilayer stack. The refractive indices of the stack layers are passed through the online optimizer to generate a new set of modified layer parameters. These parameters are then used to calculate the true optical response with the TMM analytical solver [35, 40]. After the loss is calculated, the gradients are backpropagated through the system, and the online optimizer parameters are updated. Since the matrix-based analytical solver is differentiable, it enables efficient backpropagation of gradients throughout the system, including the solver itself. The optimization results for various optical filtering spectra are given in Figure 3. The insets in the figure represent the refractive index distribution of the optimized multilayer films. For a 200-layer stack with fixed 30-nm thick layers, the optimization results in extremely close conformance with the target transmittance spectra. The largest variation between the target and the optimized device spectra occurs for the 15 nm stopband filter, shown in Figure 3(f), which can be addressed with more layers, higher refractive index ranges, and thickness optimization. To this end, the impact of the layer numbers on the performance has been investigated. Figure 4 shows the effect of the total number of layers on the design performance. For this study, the long pass and the double band stop filter spectra are chosen as the target. For both target spectra, an optimization is performed with 60 and 200-layer optical stacks with fixed layer thicknesses of 30 nm. While the performance of the long pass filters given in Figure 4(a) and (b) does not change much, dual-band stop filters given in Figure 4(c) and (d) show a significant increase in performance as the number of layers increases. Thus, it is safe to conclude that the number of layers needed for high-quality optimization increases with the increasing complexity of the target spectra.

### 4.2 Reduction of layer number

**Figure 5:**
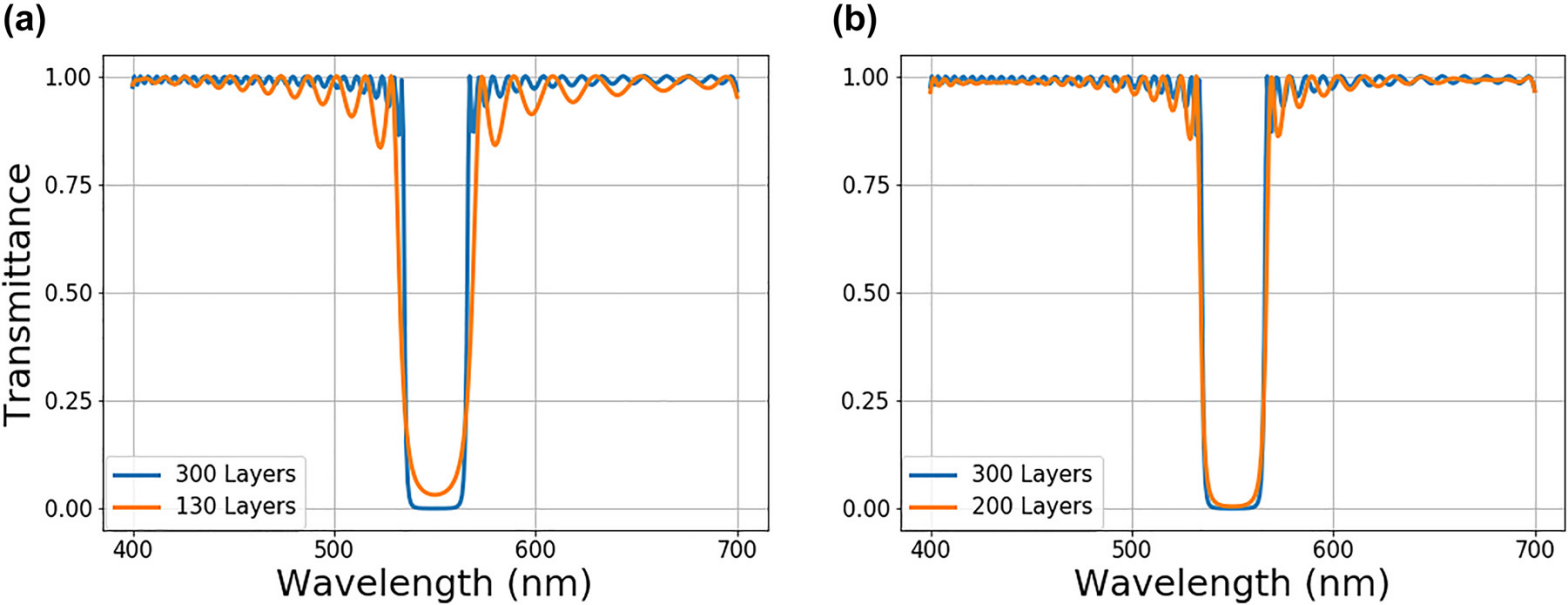
Transmittance spectra comparison between optimized stacks with different number of layers. A 300-layer optimized optical stack spectra the (orange) is optimized first. Layer numbers are gradually reduced until the stopping criterion is reached. The transmittance spectra of reduced optical stacks (blue) with (a) 130 layers, and (b) 200 layers are compared to initially optimized structure with 300 layers.

The number of layers is yet another parameter in optimizing multilayer films. Usually, the least number of layers needed to satisfy an optimization criterion is not known *a priori*. Therefore, we implement a layer number reduction scheme into the NN-based optimization architecture. For the layer number reduction, a policy of reduction and performance threshold for stopping criteria is required. As the policy of reduction, removing the last 10 layers of an optimized stack at user-defined intervals is selected in the scope of this specific aim. Different policies could also be implemented, such as combining adjacent layers with similar refractive indices. As the performance threshold, we use mean absolute percentage error (MAPE), which is defined as [41]:

$$\text{MAPE}=\frac{100}{n}\sum_{t=1}^{n}\left|\frac{T{\left(\omega_{t}\right)}_{\text{target}}-T{\left(\omega_{t}\right)}_{\text{optimized}}}{T{\left(\omega_{t}\right)}_{\text{target}}}\right|$$

where $T{\left(\omega \right)}_{\text{target}}$ and $T{\left(\omega \right)}_{\text{optimized}}$ are the target and optimized transmittance spectra. The optimization starts with a large number of layers to guarantee high performance for a given design problem. After optimization converges to a good solution, last 10 layers of the structure are removed, and the remaining structure is reoptimized. This cycle is repeated until the reduced stack can no longer satisfy the performance threshold after a reasonable number of iterations. The impact of the layer reduction is depicted in Figure 5. For the stopping criterion of MAPE 3%, the optimization started with 300 layers and reduced the structure to 130 layers. We will note that the thicknesses are fixed; therefore, the reduced structure becomes thinner. While bandstop performance is somewhat affected with 130 layers, the changes in the performance for the 200-layer structure are negligible compared to the initially optimized structure with 300 layers. In principle, one can use a weighted MAPE or different stopping criterion to prevent performance degradations within desired bands. Using the layer reduction protocol, the total number of layers becomes an optimizable parameter for multilayer optical stack design. This enables optimization with least number of layers possible, allowing an easier and cost-effective fabrication of multilayer optical films.

### 4.3 Optimization in the presence of systematic growth imperfections

**Figure 6:**
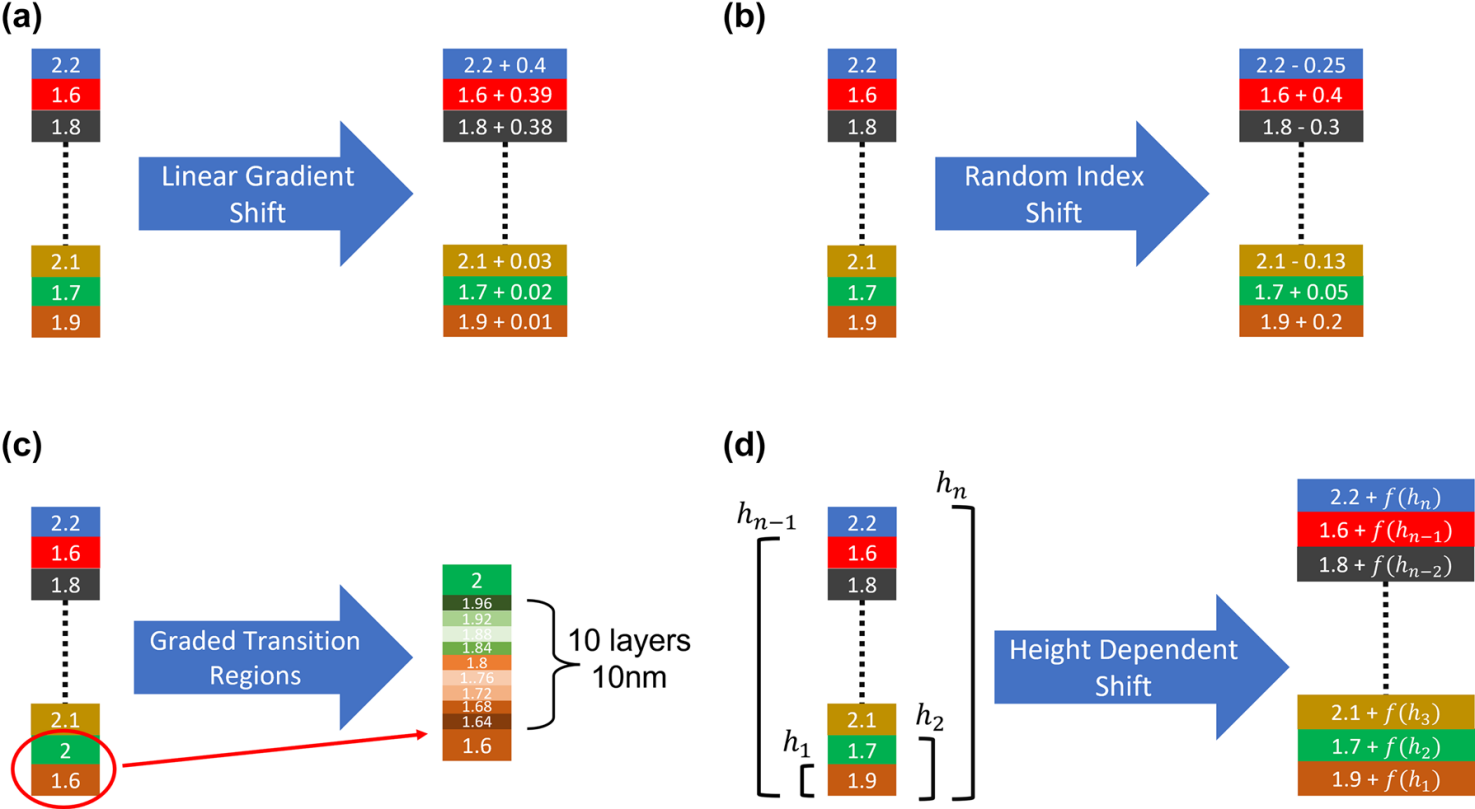
The fabrication imperfections for CVD grown single material multilayer optical stacks. (a) Linear gradient shift, (b) randomly changing shifts in refractive index, (c) graded transition regions between layers, and (d) height dependent functional shift in refractive index.

In practice, when fabricating single material continuously changing films, imperfections always occur. These imperfections could be inherent to the nature of multilayer films, such as stress-induced inhomogeneities that lead to shifts in the refractive indices. Additionally, imperfections can be inherent to growth techniques, such as imperfect chamber gas stoichiometry due to switching precursor gas ratios for each layer deposition or slow changes in precursor gasses leading to gradually changing properties instead of clean cut-offs at the layer interfaces. These imperfections will result in refractive index shifts with different dependencies and transitory regions between the layers. Accumulated over many layers, these errors can dramatically reduce the performance of the multilayer stack. Fundamentally, all fabrication imperfections are either systematic or random in their nature. In this section we will discuss systematic errors, and the random errors will follow in the next section. Systematic errors are errors that are replicated with every repeated stack fabrication. Due to their deterministic nature, these errors can be fully compensated for. A variety of systematic errors are simulated during the optimization process to understand (i) the impact of the imperfections on the performance and (ii) efficient ways to compensate for them. Specifically, we modeled a variety of possible systematic errors such as: linear gradient shifts, transitory regions between layers with refractive indices spanning from the bottom to top layer values, and height-dependent refractive index shifts in the system including polynomial, and other higher-order dependencies. As the test bed for this study, a 100-layer optical stack with refractive indices ranging from 1.6 to 2.4 and layer thicknesses ranging from 20 to 120 nm is chosen. Figure 6 depicts the systematic errors applied to each layer of the multilayer stacks. The linearly increasing index shift is implemented by linearly increasing the amount of error in the refractive index at each successive layer. The bottom layers have minimum shifts while top layers have the highest shifts of up to 0.4, which is half of the user-defined range (1.6–2.4) of 0.8. Due to the time needed to change chamber stoichiometry between layers, a transition region between layers is expected. These transitory regions are modeled as 10 nm regions centered between the layer boundaries with a linear refractive index gradient between the interface’s bottom and top layers. Specifically, the 10 nm thick transition region is divided into 1 nm thick sub-layers, with the linear shift implemented as gradual changes in the refractive indices of the sub-layers. This transitory region is applied to all layer interfaces throughout the structure. To generalize further and to demonstrate this method’s capability and versatility higher-order height-dependent functions of refractive index shift were also implemented. These functions introduce an index shift based on the layer height measured from the bottom of the multilayer stack. Specifically, the following height-dependent functions were implemented,

$$\Delta n\left(x\right)=3x^{3}-5x^{2}+2.2x-0.2$$

$$\Delta n\left(x\right)=0.2\left(\sin x^{2}+\sin3x\right)$$

$$\Delta n\left(x\right)=0.2\left(e^{\text{x}}-e^{\frac{x}{2}}-e^{\frac{x}{3}}-4x^{2}-2x\right)$$

are cubic, trigonometric and hybrid exponential-polynomial height-dependent index shift functions, respectively. $\Delta n\left(x\right)$ are the index shifts applied to a specific layer, and *x* is the distance measured from the bottom of the layer to the bottom of the multilayer stack. Note that the systematic errors will depend on the deposition tool, which is typically unknown and often difficult to measure *a priori*. The example functions are chosen to demonstrate the error compensation capability of the approach, regardless of the distribution of the systematic error.

**Figure 7:**
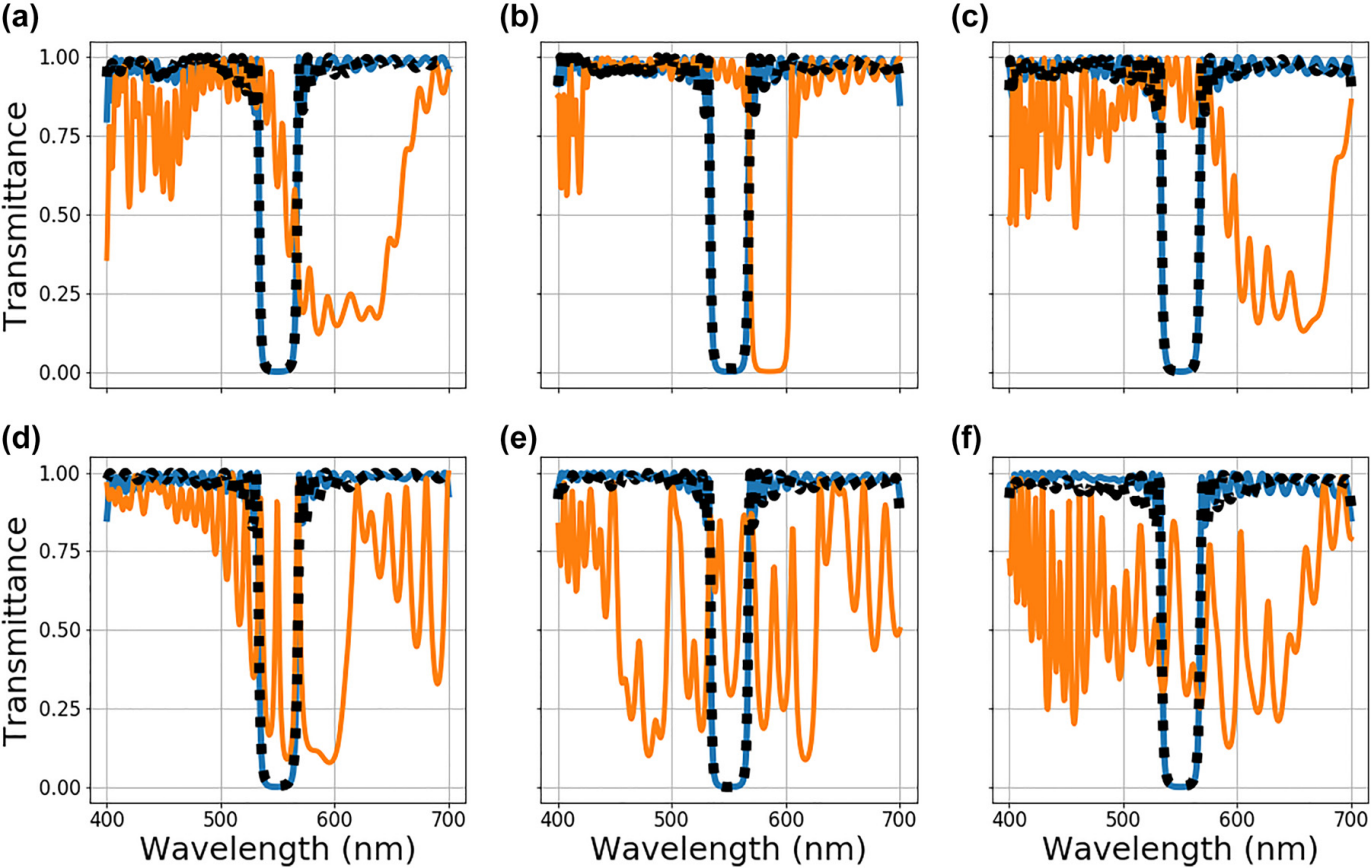
Multilayer stack optimization under deterministic fabrication imperfections. The blue curve represents the spectra of the original ideally optimized stack without any imperfection introduced. The orange curve is the spectra of the same stack but with fabrication errors added. The black dotted curve is the spectra of the stack after the second stage of optimization to compensate for the fabrication errors is complete. The different plots are for different types of systemic error introduced: (a) linear gradient shift, (b) graded transition regions between layers, (c) linear gradient shift and graded transition regions between layers combined, (d)–(f) polynomial, sinusoidal, and exponential height dependent index shifts, respectively.

These errors are implemented individually and in combination to determine the impact on the optimization performance. The results under systematic imperfections are given in Figure 7. A linear gradient shift of indices enlarges the band stop region while the entire spectrum is red-shifted, as shown in Figure 7(a). Transition regions, on the other hand, only redshift the spectra. Figure 7(c) indicates that the combination of these imperfections leads to the linear addition of the degradations introduced by them in the transmittance curve. Height-dependent index shift functions depicted in Figure 7(e)–(f) yield more significant performance degradation, which is more fundamental than a simple red/blue shift. Based on these results, it is safe to conclude that systematic errors can significantly impact the optimized stack performance.

**Figure 8:**
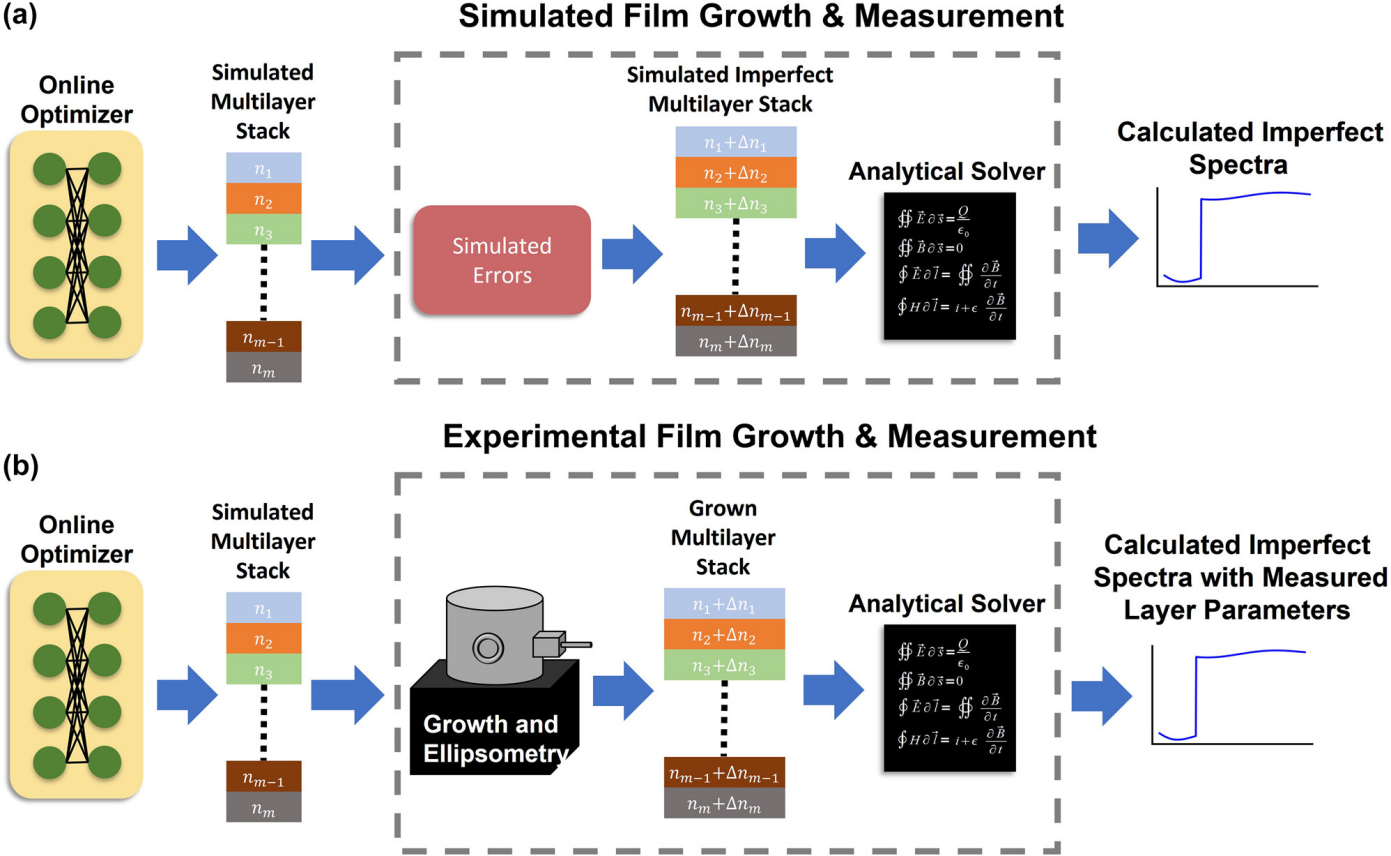
Network architecture for fabrication-in-loop NN-based inverse design of single-material multilayer optical stacks with continuously changing refractive index. (a) The optimization starts under ideal conditions and iterates until convergence. Later, fabrication-related imperfections are added to the ideally produced stack to simulate the imperfections from deposition tools. Modified stack response is calculated and the loss is backpropagated to update NN parameters. This procedure simulates the practical fabrication, (b) case. After ideal optimization, optimized parameters are used to grow the desired multilayer film. The imperfectly grown film will be measured and experimentally retrieved layer parameters are integrated back into the optimization cycle to calculate the imperfect response and loss function. This allows direct integration of imperfections related to the growth process into the optimization loop without disrupting the backpropagation chain. Simulated errors replace the film growth and ellipsometry in the optimization process for the experimental realization of the multilayer films.

A fabrication-in-loop inverse design approach is proposed to counteract the systematic errors in the film growth process effectively. The details of the approach are shown in Figure 8. The proposed method consists of two phases. In the first phase, a multilayer optical stack is optimized with the NN-based inverse design architecture given in Figure 2. The resulting high-performing multilayer film design is then fabricated in the second phase. During the growth process, layer parameters are extracted with in-built metrology tools, which are available in many deposition machines. Specifically, after each layer is grown its properties are extracted with ellipsometry layer-by-layer [14]. Once the imperfect layer data is extracted from experimental measurements, they are integrated into the optimization cycle. To achieve this, the experimentally measured layer parameters are passed through the analytical solver to calculate the expected transmittance spectra. The resulting loss function is then calculated with the imperfect response. This approach allows a gradient-based inverse design method such as the one proposed here to effectively learn the response of a so-called “black box” system, which is the deposition tool in this case. In essence, by using the experimental layer parameters in the optimization cycle, imperfections in the deposition tool can be learned/compensated with a gradient-based optimization method. Importantly, the backpropagation chain is continuous, which is necessary for NN training. Consequently, the NN-based inverse design must learn the imperfections of the machine to produce high-quality stacks in addition to the general EM response of the multilayer films. This cycle of fabrication, measurement, backpropagation, and update continues until a high-performance device is realized experimentally.

In the scope of this study, the experimental growth and measurement process is simulated with systematic fabrication imperfections, as shown in Figure 8(a). After convergence to a good solution in the first phase, the selected error function is applied, and the refractive indices of individual layers are modified accordingly in the second phase. The modified stack response is calculated with the analytical solver, and the gradients are backpropagated after loss function calculation. This procedure, shown in Figure 8(a), simulates the practical fabrication cycle, as shown in Figure 8(b). We note that the introduced errors are not static, meaning that the error that depends on the layer properties, such as index and thickness, are changed every iteration with different optimized stacks.

The results of the compensation for fabrication imperfections are shown in Figure 7 (black dotted line). In the second phase of the optimization, starting from the modified stack with an imperfect response (solid orange line), the NN-based inverse design scheme learns to compensate for fabrication errors to a high degree. As a result, it produces a stack specifically modified to offset the impact of the fabrication errors for all the types of systematic error studied. Analyzing this procedure, the results indicate that the systematic errors in a deposition device can be fully accounted for in the optimization loop to realize high-performance multilayer stacks. The Supplementary Figures S2–S7 shows the loss track and modified device performance in intermediate steps. These results indicate that depending on the complexity of the device, from few tens up to a hundred cycles of optimization can yield multilayer stacks with high performance. The number of iterations also depends on the severity of the errors in the system, the learning rate, and the neural network architecture, which can be optimized for a given design problem. We would like to note that the NN-based inverse design proposes a modified stack, which offsets the imperfections introduced by the deposition tool. In an error free fabrication process, the multilayer film grown with error-compensated layer parameters will perform poorly as the NN is trained to change the layer parameters with respect to the fabrication imperfections. Naturally, the multilayer stack can be optimized with fabrication in the loop from the beginning as shown in Figure 8(b). However, this has the effect of increasing the number of optimization iterations with time consuming experimental growths required as indicated by results shown in Figures S2-S7 in the Supplementary text. This unsurprising result stems from the fact that the NN is learning the EM response of the multilayer film stack in addition to the behavior of the fabrication process in this case. Thus, our two-stage fabrication-in-loop inverse design method minimizes the number of optimization iterations with time-consuming and expensive experimental growths required.

### 4.4 Optimization in the presence of random growth imperfections

Besides systematic errors, random and unrepeatable errors could be present in the deposition of multilayer films. These errors change from one fabrication process to another and are characterized statistically. Since truly random effects cannot be compensated, they pose a challenge in producing high-quality multilayer films. As pointed out in the introduction, robustness schemes such as including refractive index and layer thickness sensitivities in the loss term of the multilayer film optimization have been proposed to alleviate the impact of the random imperfections [15], [16], [17], [18], [19, 42, 43]. However, these methods have shown limited improvements against minor imperfections where random thickness changes are predicted to be less than a nanometer. This section extends the fabrication-conscious optimization approach to include the case of stochastic imperfections. Following the previous section, the test bed for this study is a 100-layer optical stack with layers having refractive index and layers thicknesses ranging from 1.6 to 2.4 and 20–120 nm, respectively. Following the architecture outlined in Figure 8, the first stage of the optimization produces a high-performing multilayer stack assuming perfect fabrication. In the second stage, simulated random errors are added to the ideal layer parameters, and the EM response is calculated with the imperfect stack, as shown in Figure 8(a). The details of the backpropagation with random imperfections in the optimization loop are given in the Supplementary materials. At every iteration, a different set of random perturbations to both index and thickness is added to the ideal stack parameters. The optimization cycle with random imperfections is ended manually after 700 iterations. Rigorously, we implement the random fabrication errors as additive noise to the learnable base of multilayer stack parameters which can be expressed as:

$$n_{\text{imp}}=n_{\text{id}}+n_{\text{id}}n_{\text{rand}}↔n_{\text{rand}}=2\gamma U\left(0,1\right)-\mu$$

$$t_{\text{imp}}=t_{\text{id}}+t_{\text{id}}t_{\text{rand}}↔t_{\text{rand}}=2\gamma U\left(0,1\right)-\mu$$

**Figure 9:**
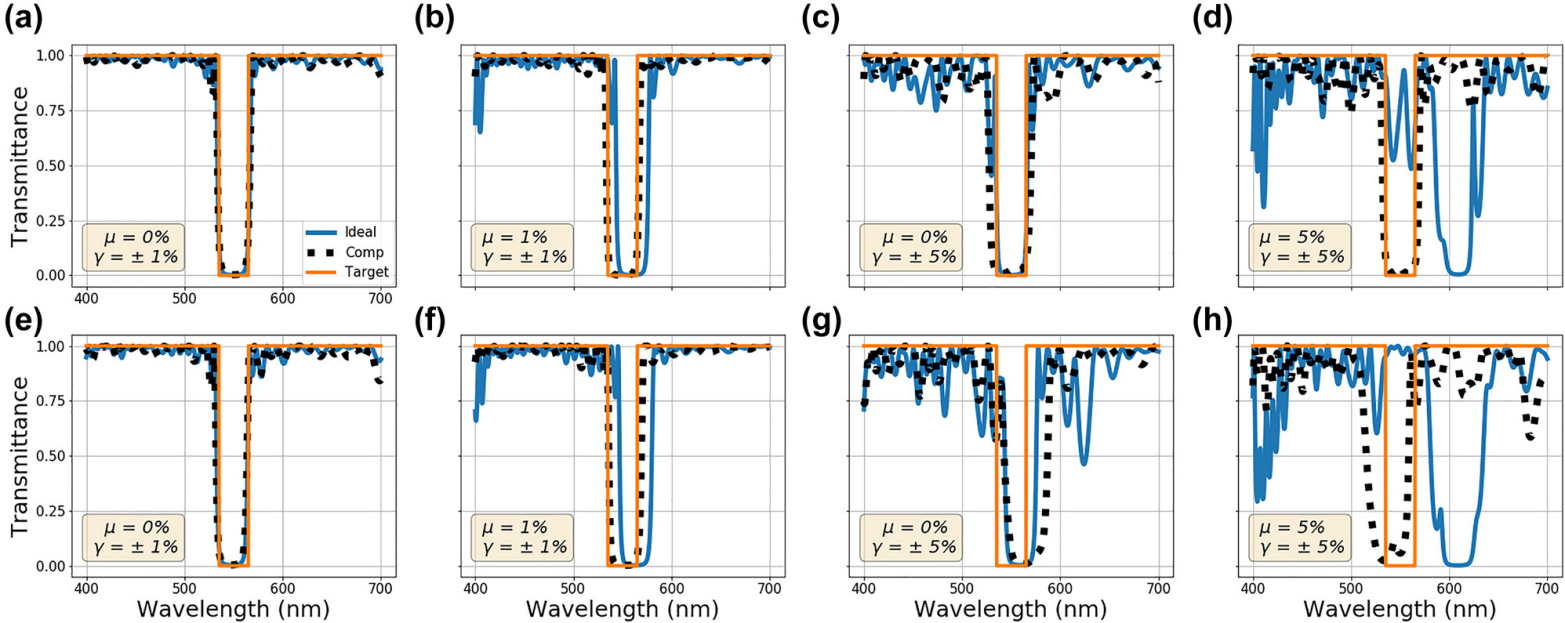
Multilayer stack optimization in the presence of random fabrication imperfections. Results after random error is added for both ideally optimized stacks (blue solid line) and stacks optimized considering random imperfections during the optimization process (black dotted line). The orange solid line is the target spectra. The transmittance of the multilayer stacks produced by both methods is tested with 1000 different sets random imperfections. The transmittance curves for the highest and the lowest performing stacks from their respective sets of results are shown in the top (a)–(d) and bottom (e)–(h) rows, respectively. The mean and the range of the uniform distribution used to create random imperfections are (a and e) *μ* = 0% and *γ* = ±1%, (b) and (f) *μ* = 1% and *γ* = ±1%, (c) and (g) *μ* = 0% and *γ* = ±5%, and (d and h) *μ* = 5% and *γ* = ±5%, respectively.

The imperfect layer parameters *t*_imp_, *n*_imp_ are obtained by adding random error functions *t*_rand_, *n*_rand_ to the ideal layer parameter *t*_id_, *n*_id_. $U\left(0,1\right)$ is a random variable with a uniform distribution between 0 and 1, and *γ* and *μ* are the user-defined error rate range and the mean of the error distribution, respectively. The results of the optimization with stochastic fabrication imperfections are given in Figure 9. To allow for a fair comparison between multilayer stacks optimized taking into account random imperfections during optimization (black dotted line) and those optimized assuming no imperfections (blue solid line), the EM response of multilayer stacks obtained with both methods have been calculated for 1000 different sets of random perturbations. Figure 9(a)–(h) depict the highest and the lowest performing samples from their respective sets according to the calculated mean squared loss for the given target spectra (solid orange line). For a mean error rate (*μ*) of 0 and error rate range (*γ*) of 1 percent, multilayer stack optimized with and without random perturbations perform quite similarly and overall performance is not affected for the best and worst cases as seen in Figure 9(a) and (e), respectively. For *μ* = 1% and *γ* = ±1%, a red shift in the spectra is observed for the ideally optimized stack, while the stack optimized with random perturbations compensates for this effect, as shown in Figure 9(b) and (f). For *μ* = 0% and *γ* = ±5%, the impact of the random error is more significant as shown in Figure 9(c) and (g). Both stacks perform similarly for best and worst cases with minor improvements in the bandpass region for the fabrication-conscious design. Similar to Figure 9(b) and (f), the case of random error with non-zero mean, *μ* = 5% and *γ* = ±5% shown in Figure 9(d) and (h) indicates a redshift for the ideally optimized stack with an even greater impact on the performance. Regardless of the mean of the random error, optimizations with random imperfections perform similarly for the same error ranges. We thus conclude that the proposed NN-based inverse design method can effectively learn the random error’s mean and compensate for it. Unsurprisingly, random imperfections are not fully compensated, as seen in Figure 9, where the impact of the stochastic imperfections remains even for a multilayer stack optimized with fabrication-conscious NN-based inverse design.

**Figure 10:**
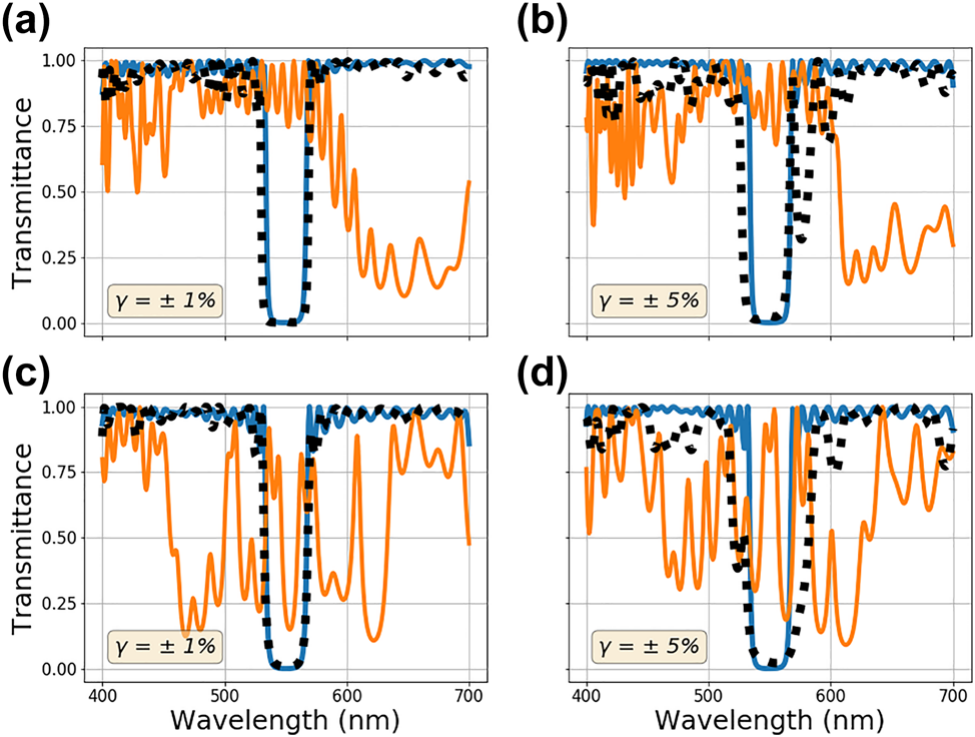
Multilayer stack optimization in the presence of both deterministic and random fabrication imperfections. (a) and (b) Linear gradient shift and graded transition regions between layers combined with *γ* = ±1% and *γ* = ±5% random index shifts, respectively (c) and (d) sinusoidal height dependent index shifts (2) with *γ* = ±1% and *γ* = ±5% random index shifts, respectively. The solid blue curve represents the ideally optimized spectra without any error. The orange curve represents the ideally optimized spectra after both the error types are introduced. The black dotted curve is the result of optimization to compensate for the errors.

Finally, the impact of combined random and systematic errors is investigated, and the results are shown in Figure 10. For the bandstop filter design, 2 sets of systematic and random errors are implemented. The mean of the stochastic error rate for all the results shown in Figure 10 is 0 percent (*μ* = 0%). Linear grading and transitory regions are applied with *γ* = ±1%, and *γ* = ±5% random shifts to each layer. The results are shown in Figure 10(a) and (b), respectively. The results for height-dependent sinusoidal function with a *γ* = ±1%, and *γ* = ±5% random shifts are shown in Figure 10(c) and (d). Looking at the figures and related loss tracks in Figures S2–S7 of the Supplementary, it is clear that the systematic errors are learned and compensated for even in the presence of random errors. Comparing the results with corresponding random error rates between Figures 9 and 10, it is clear that full compensation of the deterministic errors is achievable even in the presence of random imperfections. Starting with both random and deterministic imperfections (solid orange line), a multilayer stack optimized using the fabrication-conscious approach outlined earlier can reach performance only limited by the random imperfections (black dotted line). This approach is most advantageous when systematic errors are dominant in the growth process. Essentially, regardless of the nature of the systematic error, this fabrication-in-loop optimization scheme can produce high-performance films even in the presence of stochastic deposition errors.

## 5 Conclusions

As the complexity of photonics design requirements grow, the need for novel and effective fabrication conscious inverse design techniques increases. This work proposes an NN-based inverse design scheme for single-material variable index multilayer films. Additionally, we demonstrated an approach using our NN-based inverse design technique to compensate for fabrication imperfections introduced by the multilayer film growth into the optimization process. The automatic differentiation capability provided by the differentiable analytical solver used enables efficient integration of NNs into the optimization scheme. This procedure requires no user intervention or physics knowledge of the problem on the user’s part. Under ideal conditions, the NN-based inverse design scheme produces multilayer structures with nearly ideal spectra. These designs were produced with a single material (Si_x_N_y_), with a continuously variable index. The refractive index variability was taken from the literature to ensure the experimental feasibility of this approach. We also demonstrated a method to reduce the number of layers without significantly sacrificing performance for fabrication simplicity. Finally, a fabrication-in-loop design technique has been proposed to compensate for the errors resulting from the fabrication process. The imperfections introduced during deposition are simulated as layer refractive index and thickness variations. These variations can either be systematic or random errors and both classes were analyzed in this work. By simulating the imperfections and including them during a second stage of neural network training, we showed that the systematic errors could be compensated for resulting in designs that perform ideally even in the face of significant systematic error. For the random errors, our results indicate the proposed method can learn the fundamental characteristics of the random distribution and correct for spectra shifts. Furthermore, our results conclusively show that systematic errors can be learned and compensated for, even in the presence of significant random errors.

In practice, the simulated imperfect deposition would be replaced with actual deposition and characterization of the resulting stack. The outlined method allows a gradient based optimizer to work with a so called “black box system” in the optimization cycle by retrieving layer parameters during the growth process. In principle, one can avoid the measurement during the growth by utilizing gradient-free approaches such as particle swarm optimization, genetic algorithm, deep reinforcement learning, et cetera. However, gradient-based approaches converge faster due to the additional information given by the local gradients of the system, provided that they are not stuck on a local minimum. The faster convergence is crucial as it minimizes the number of expensive fabrication cycles required. Naturally, a possible extension of this study is to explore methods utilizing gradient-free optimization methods in such a way that the necessary number of optimization cycles is practically achievable. In future work, the second stage optimization of the neural network will be done using real experimental data. Importantly, this study demonstrated that the number of required samples to perform the second stage training of the network is relatively small (30–100) meaning that this approach is experimentally practical. This fabrication-oriented design approach paves the way towards the realization of robust high-performance single-material index variable multilayer films. With their superior optical, thermal, and mechanical properties, we believe these structures will play more significant role in future optical applications. Particularly, as design techniques such as those outlined in this paper address the practical fabrication difficulties associated with the realization of such devices.

## Supplementary Material

Supplementary Material Details
