# Supplementary material for: Fabrication-conscious neural network based inverse design of single-material variable-index multilayer films
Source: Nanophotonics. 2023 Jan 30;12(5):993–1006. doi: 10.1515/nanoph-2022-0537 (PMC11501231; doi:10.1515/nanoph-2022-0537)
Supplement: Supplementary file 1 — Supplementary Material Details [file j_nanoph-2022-0537_suppl.docx]

Omer Yesilyurt*, Samuel Peana*, Vahagn Mkhitaryan, Karthik Pagadala,
Vladimir M. Shalaev, Alexander V. Kildishev, and Alexandra Boltasseva

Fabrication-conscious Neural Network Based Inverse Design of Single-Material Variable-Index Multilayer Films: Supplementary Materials

1. Neural Network-Based Inverse Design vs. Stochastic Gradient Descent Optimization for Multilayer Optical Film Design

Automatic calculation of local gradients enables high-performance optimization for many physics problems. Depending on the complexity of the problem, simpler optimization protocols could perform similarly to complex methods when local gradients are available. Here, we compare a direct stochastic gradient-based (SGD) optimizer to an NN-based optimization. Specifically, Adaptive Moment Estimation (Adam) is chosen as the direct SGD optimizer. It is a first-order gradient-based optimization based on adaptive estimates of lower-order moments [1]. Adam is also used to optimize the weights and biases of the NN, allowing a fair comparison and clear demonstration of the impact of the NN. The optimization target is a bandpass filter with a passband of 437.5-475 nm (a solid green line in Fig. S1).


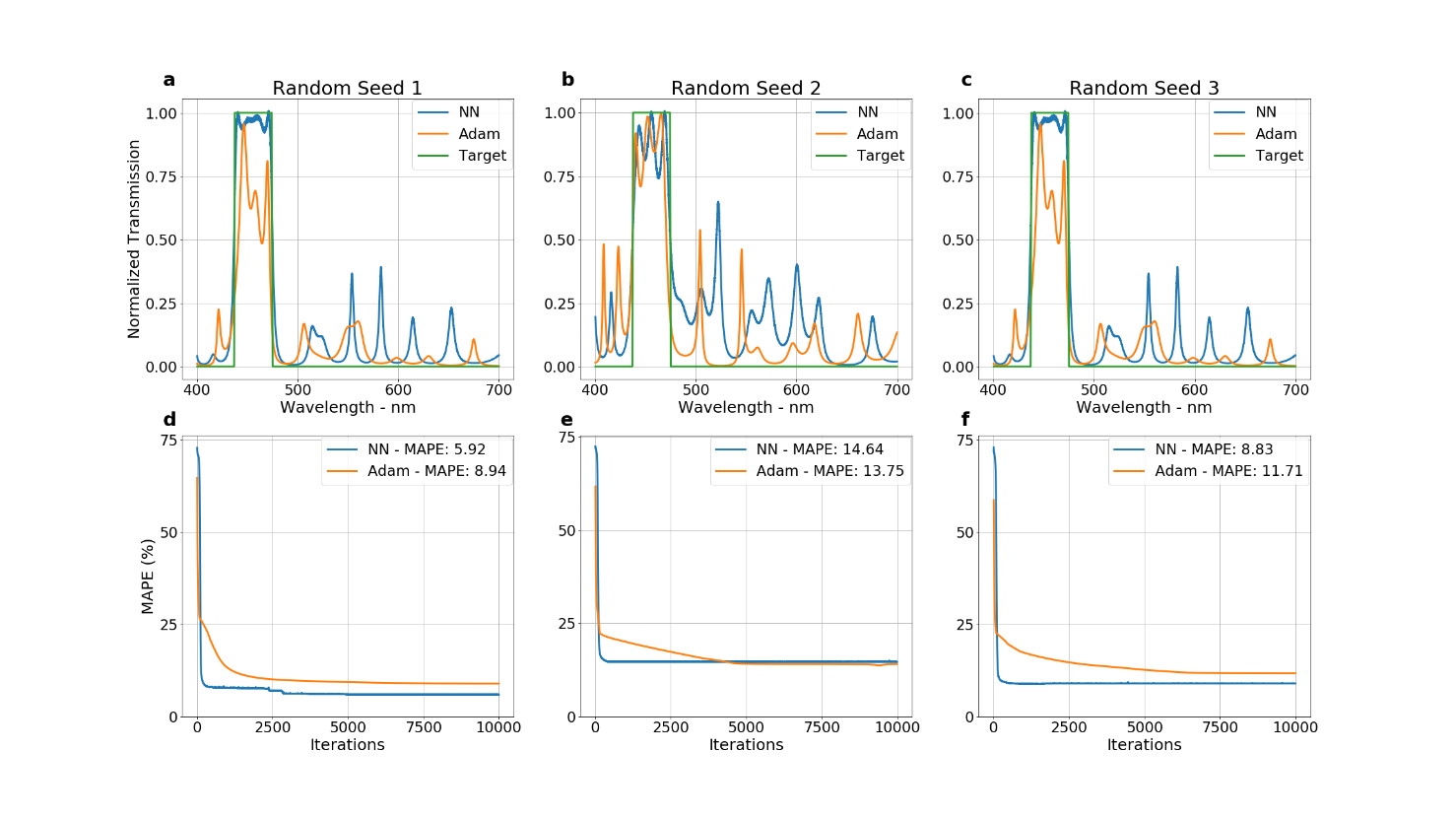


Figure S1. Comparison of NN-based vs direct SGD optimizer for multilayer optical film optimization. (a, b, c) Transmittance spectra comparison between the NN (blue) and Adam (orange) optimized optical stack spectra for a band pass filter with 437.5 – 475 nm passband. The target spectrum is shown with a solid green line. (d, e, f) Convergence comparison of the mean absolute percentage error (MAPE) for NN (blue) and Adam (orange) optimized optical stack. The legend also shows the lowest MAPE for each curve in the respective plots.

Both methods employ 30 layers, 1.6 to 2.4 refractive index range, and 20- to 200-nm thickness range to optimize a multilayer film for the given target. The optimization is stopped after the 10,000^th^ iteration. The learning rate is instrumental in optimizing the convergence speed. While small update steps may slow down the convergence significantly, large steps may cause instabilities in the solution search and result in non-convergence. A parameter sweep for the learning rate is performed to make a fair comparison of the convergence speed. Empirically, we found that the learning rate of 1e-5 is a good but slow converging starting point for both methods. We increased the learning rate gradually until failure for both methods. The results for the fastest convergence learning rates are given in Figure S1. The same optimization procedure is performed with different random seeds three times to show the impact of the initialization on the optimization. Both methods are given the same input structures for the different random seeds.

Figures S1 a-c depict the spectra multilayer stacks optimized with the NN-based approach and Adam optimizer. While their out-of-band spectra are comparable in performance, passbands are visibly better for NN-based inverse-designed multilayer stacks for all random seeds. The convergence of mean absolute percentage error (MAPE) for the optimized spectra is given in Figures S1 d-f. NN-based optimization converges up to 2 orders of magnitude faster compared to Adam.

We note that the speed advantage shown here may change with the problem complexity, available optimization resources, and the type of gradient/gradient-free optimization techniques. However, our results indicate that for the given problem, NN-based optimization could offer remarkable speed-up, achieving comparable or better performance. The fundamental difference between the two methods presented here comes from the content of their optimization hyperplanes. In the direct SGD optimizer, the layer properties constitute the optimization hyperplane, while the same SGD approach optimizes the neural network parameters in the NN-based optimization. Since the number of the NN parameters is significantly larger than the layer parameters, it seems counterintuitive that NN based approach converges faster with a much larger parameter space to optimize. However, it has been shown both empirically and theoretically for some cases that direct SGD optimizers converge faster in the overparametrized loss landscape of NNs. Gradient descent based optimization schemes are known to perform poorly in non-convex systems. Typically, loss landscape of an overparametrized NN is not convex, even locally. However, it has been shown that the non-convex loss landscape of the overparametrized NNs stasify Polyak- Lojasiewicz condition for most of the parameter space. Systems that satisfy this condition have solutions which can be accessed efficiently with gradient descent based optimizers [2]. We refer the reader to [2]–[7] for a detailed analysis on the impact of overparameterization; this effect is beyond the scope of this study. Qualitatively, we surmise that the overparameterization introduced by the NNs enables the speed-up for multilayer film optimization.

1. Multilayer Optical Film Optimization with Deterministic and Random Errors

In this section, we present a more detailed look at the optimization of multilayer films with fabrication imperfections. Figures S2-7 show the transmission spectra of the multilayer films after the introduction of the errors at chosen intervals. Specifically, first 300 iterations of the optimization are performed under ideal conditions. Errors are introduced at the 301^st^ iteration and the optimization continues another 300 iterations under the effect of imperfections. The impact of the error introductions is clearly shown with a spike in the loss tracks given Figures 2-7 (f). These spikes at the 301^st^ iteration is considered as the first iteration of the optimization with fabrication imperfections. The iteration numbers in Figures 2-7 (a-e) refer to iterations after the error introduction. In their respective loss track plots given in Figures 2-7 (f), the spectra shown in Figures 2-7 (a-e) are highlighted with red points. A steady decrease in the loss clearly indicates that the NN is effectively learning the systematic errors and compensating for them. Figures S2 and S3 depicts the results for systematic errors which are linear grading and transitory regions and sinusoidal height dependent index shifts, respectively. Figures 4-7 shows the results for the same deterministic errors in combination with random index shifts. The results for linear grading and transitory regions with $\gamma=\pm1\%$ and $\gamma=\pm5\%$ random index shifts are shown in Figures S4 and S6, respectively. Same results for height dependent index imperfections with $\gamma=\pm1\%$ and $\gamma=\pm5\%$ random index shifts are shown in Figures S5 and S7, respectively.


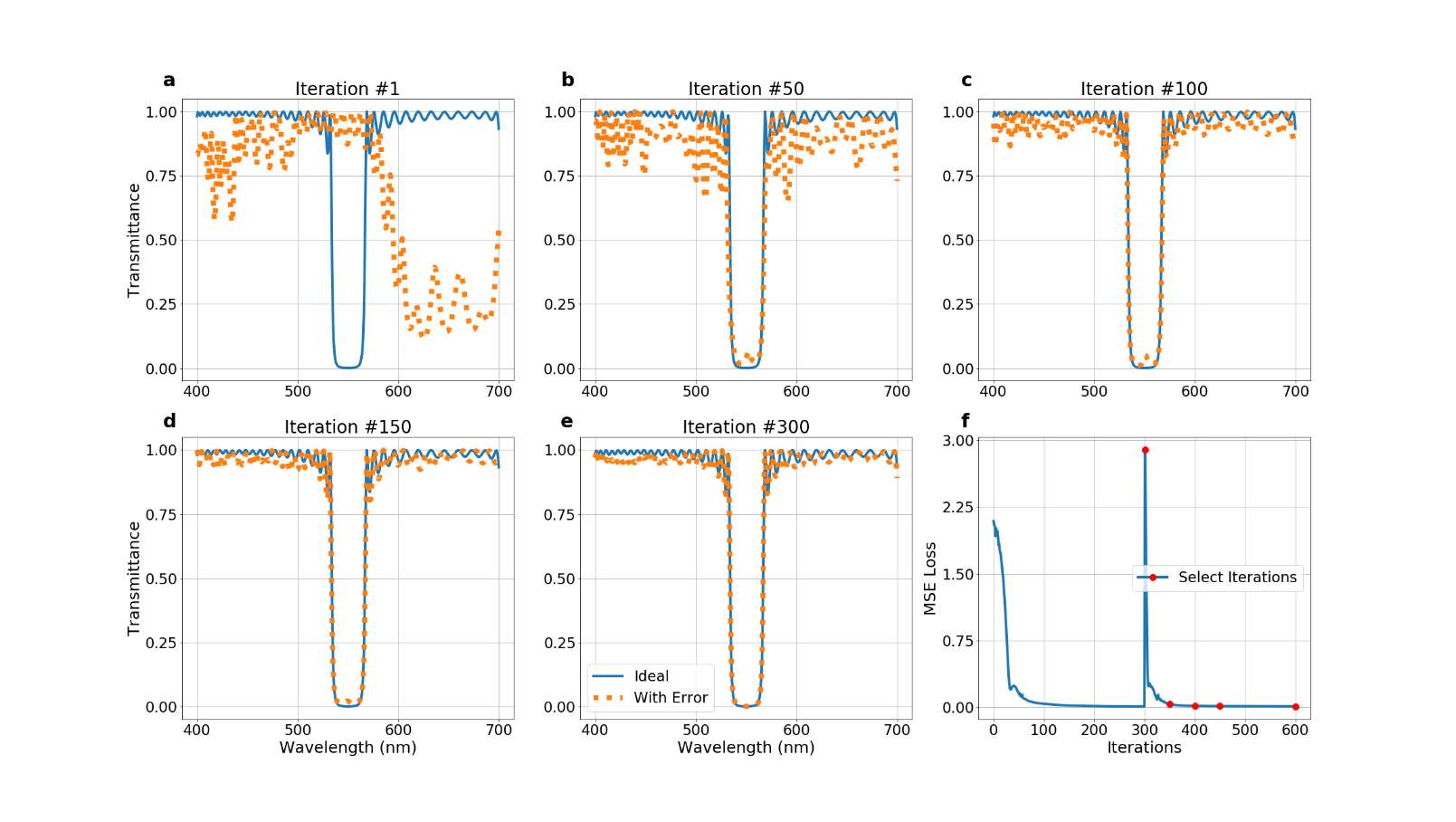


Figure S2. Evolution of the optimized spectra after the error introduction. The ideally optimized spectrum (blue solid line) is compared to the spectra multilayer film optimized with linear grading and transitory regions imperfections (orange dotted line) at (a, b, c, d, e) 1^st^ ,50^th^ ,100^th^, 150^th^ and 300^th^ iterations, respectively. The loss track (f) indicates how close are the target and optimized spectra for a given iterations. The error introduction takes place at 300^th^ iteration. Red points highlight the losses of the iterations shown in (a, b, c, d, e), respectively.


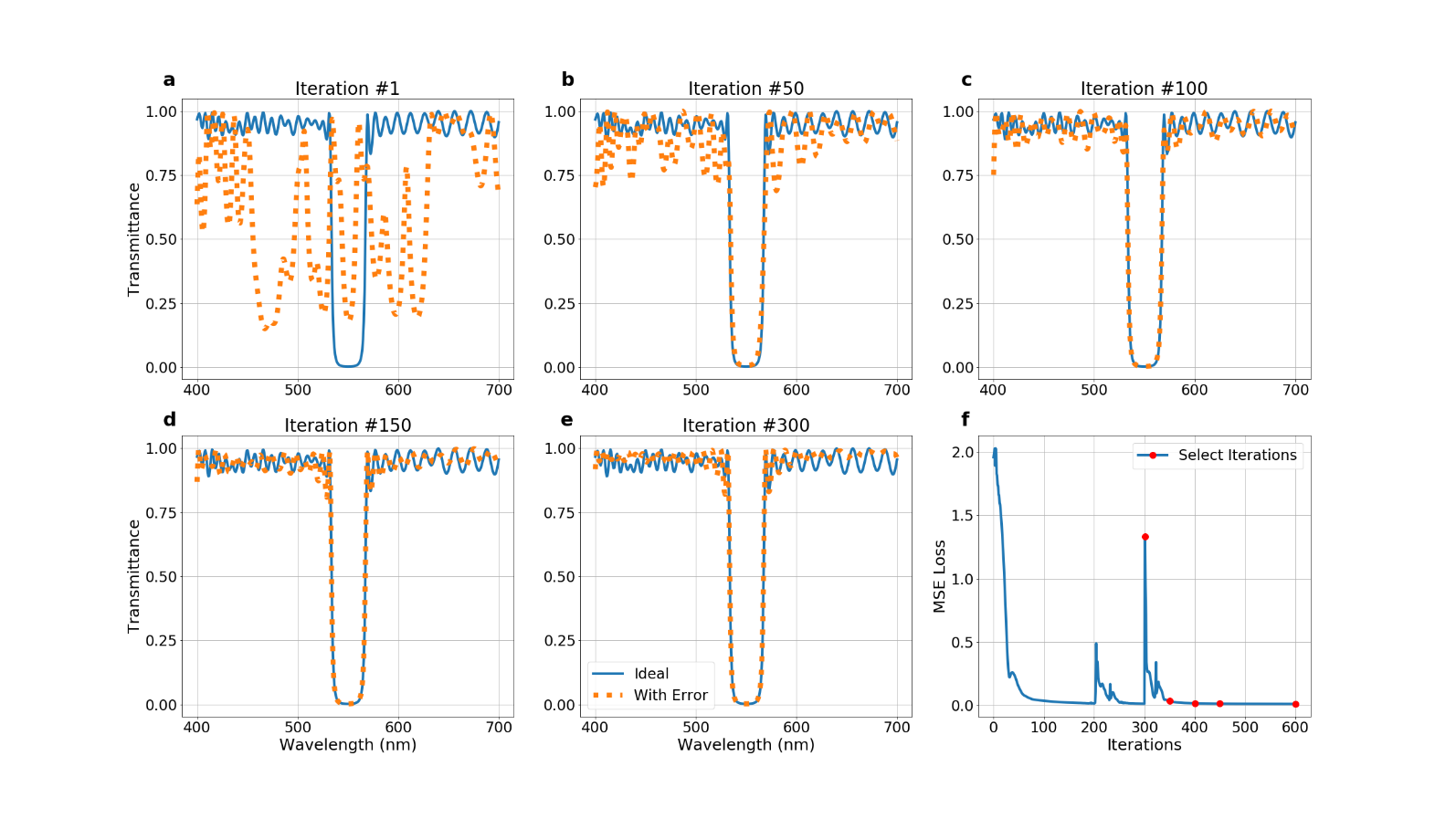


Figure S3. Evolution of the optimized spectra after the error introduction. The ideally optimized spectrum (blue solid line) is compared to the spectra multilayer film optimized with sinusoidal height dependent imperfections (orange dotted line) at (a, b, c, d, e) 1^st^ ,50^th^ ,100^th^, 150^th^ and 300^th^ iterations, respectively. The loss track (f) indicates how close are the target and optimized spectra for given iterations. The error introduction takes place at 300^th^ iteration. Red points highlight the losses of the iterations shown in (a, b, c, d, e), respectively.


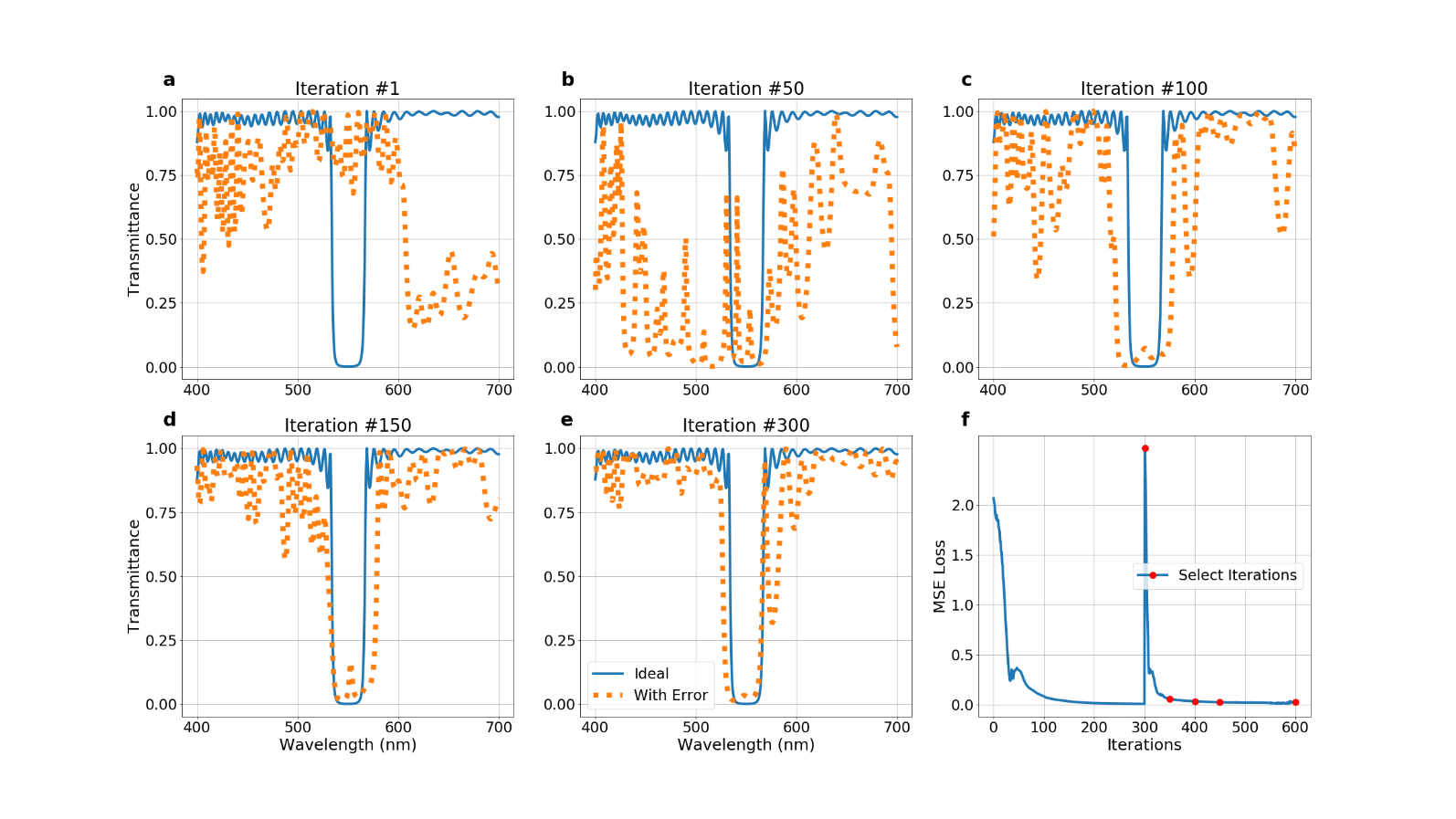


Figure S5. Evolution of the optimized spectra after the error introduction. The ideally optimized spectrum (blue solid line) is compared to the spectra multilayer film optimized with sinusoidal height dependent and $\gamma=\pm1\%$ random index shift imperfections (orange dotted line) at (a, b, c, d, e) 1^st^ ,50^th^ ,100^th^, 150^th^ and 300^th^ iterations, respectively. The loss track (f) indicates how close are the target and optimized spectra for a given iterations. The error introduction takes place at 300^th^ iteration. Red points highlight the losses of the iterations shown in (a, b, c, d, e), respectively.


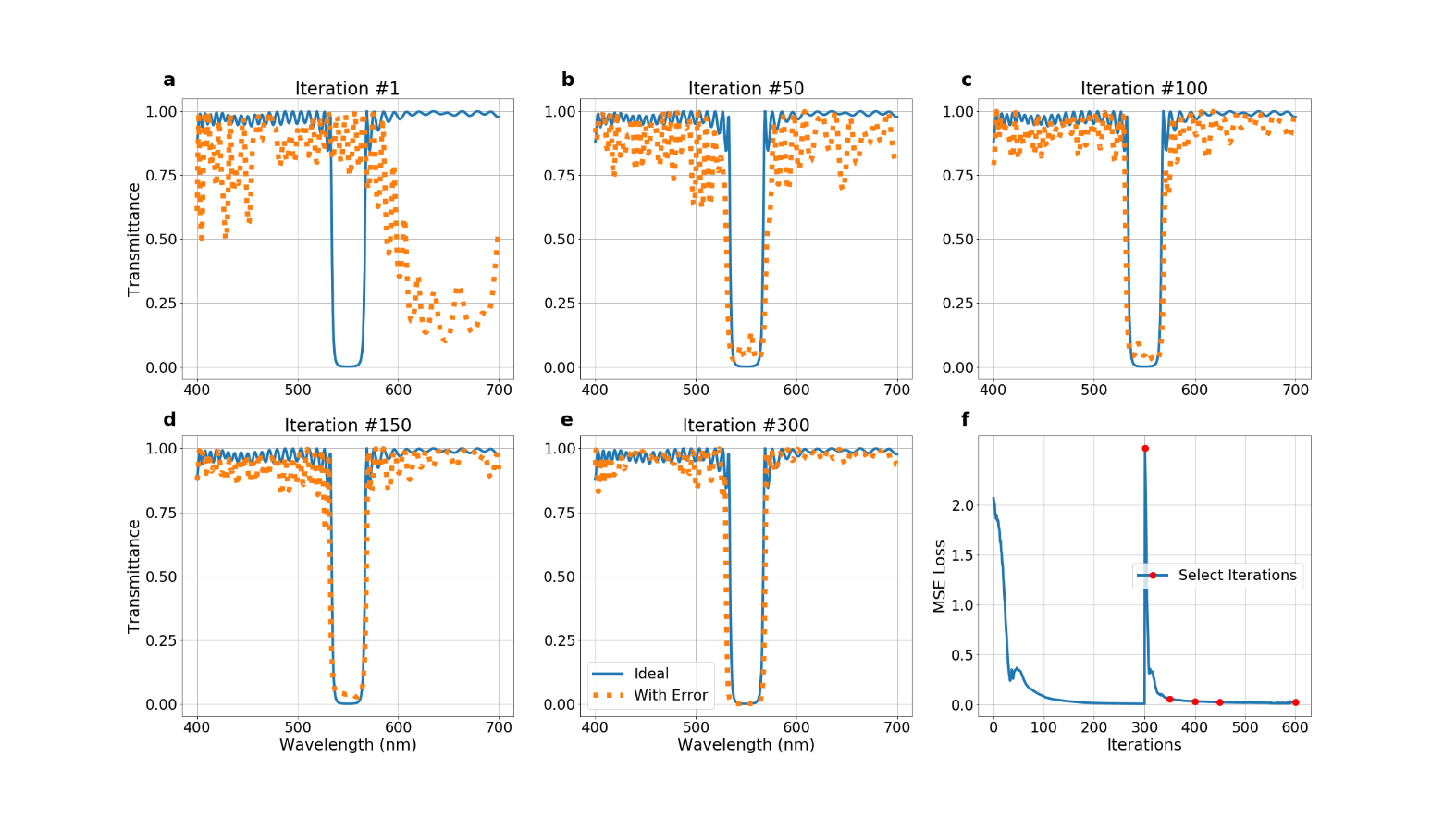


Figure S4. Evolution of the optimized spectra after the error introduction. The ideally optimized spectrum (blue solid line) is compared to the spectra multilayer film optimized with linear grading, transitory regions and $\gamma=\pm1\%$ random index shift imperfections (orange dotted line) at (a, b, c, d, e) 1^st^ ,50^th^ ,100^th^, 150^th^ and 300^th^ iterations, respectively. The loss track (f) indicates how close are the target and optimized spectra for a given iterations. The error introduction takes place at 300^th^ iteration. Red points highlight the losses of the iterations shown in (a, b, c, d, e), respectively.


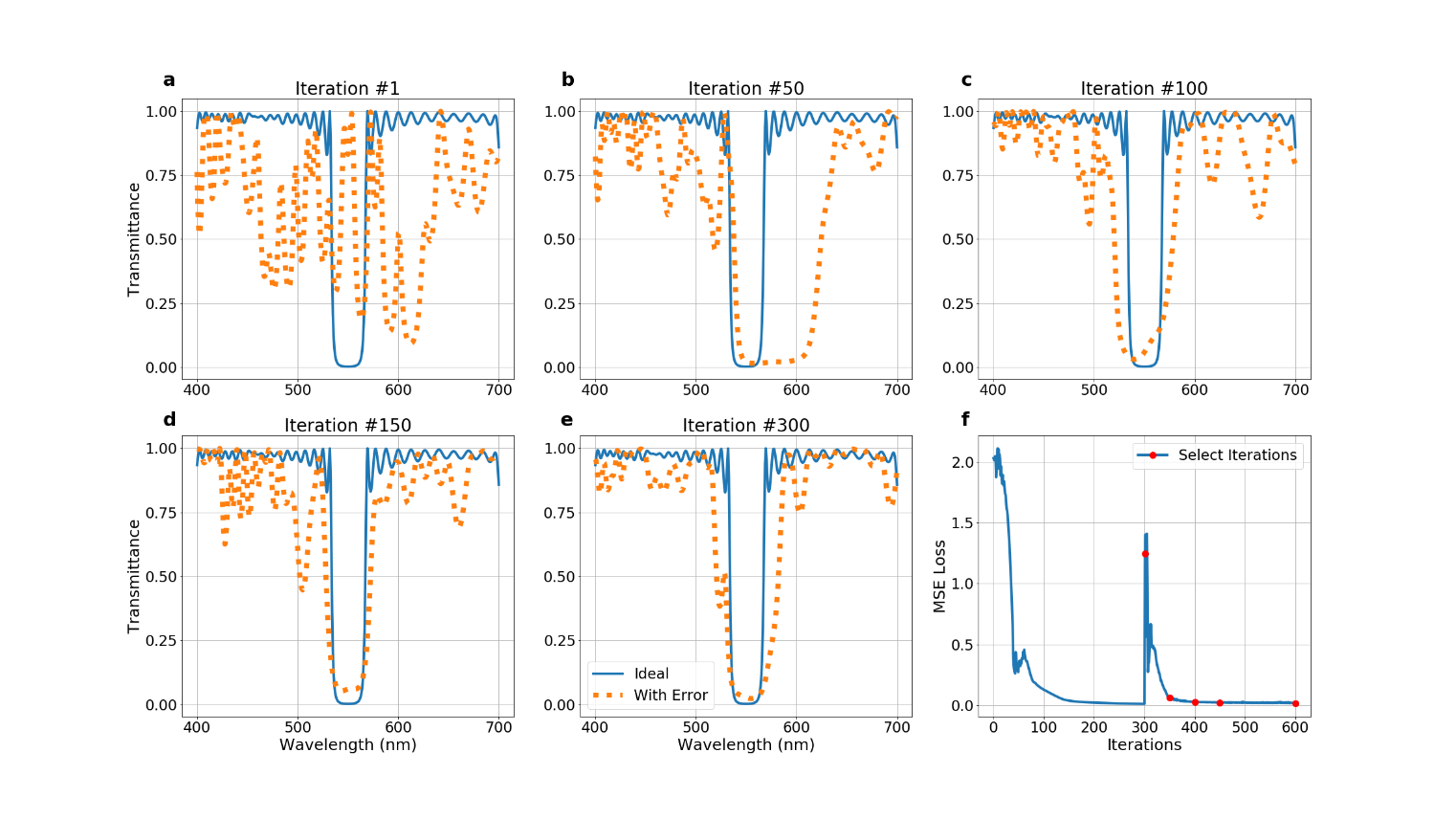


Figure S7. Evolution of the optimized spectra after the error introduction. The ideally optimized spectrum (blue solid line) is compared to the spectra multilayer film optimized with sinusoidal height dependent and $\gamma=\pm5\%$ random index shift imperfections (orange dotted line) at (a, b, c, d, e) 1^st^ ,50^th^ ,100^th^, 150^th^ and 300^th^ iterations, respectively. The loss track (f) indicates how close are the target and optimized spectra for a given iterations. The error introduction takes place at 300^th^ iteration. Red points highlight the losses of the iterations shown in (a, b, c, d, e), respectively.


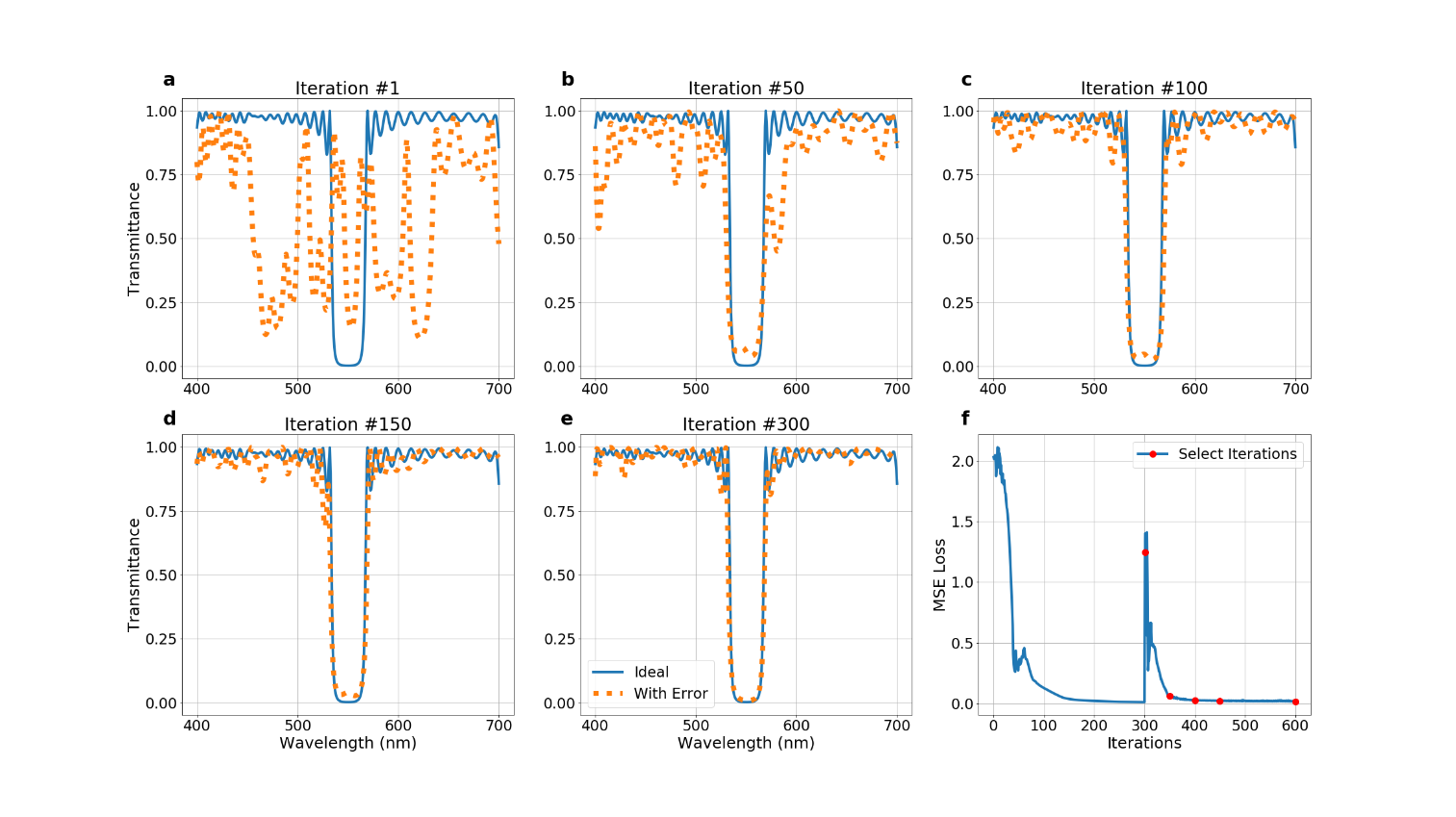


Figure S6. Evolution of the optimized spectra after the error introduction. The ideally optimized spectrum (blue solid line) is compared to the spectra multilayer film optimized with linear grading, transitory regions and $\gamma=\pm5\%$ random index shift imperfections (orange dotted line) at (a, b, c, d, e) 1^st^ ,50^th^ ,100^th^, 150^th^ and 300^th^ iterations, respectively. The loss track (f) indicates how close are the target and optimized spectra for a given iterations. The error introduction takes place at 300^th^ iteration. Red points highlight the losses of the iterations shown in (a, b, c, d, e), respectively

1. Neural Network-Based Optimization with Random Errors for Multilayer Optical Films

Random errors may be present in the multilayer optical film growth process. Generating robust designs minimally affected by random fabrication imperfections requires incorporating these random effects into the optimization process. As the main text highlights, our inverse design method is based on NN training with backpropagation. However, having a stochastic node in the backpropagation path due to the incorporation of random growth imperfections prevents backpropagation of the gradients since the derivative of a random variable cannot be calculated. Traditionally, a similar problem is encountered in variational autoencoders[8]. By changing variables, the reparameterization trick can break down a random variable into its deterministic base and random components. In our implementation of the random imperfections to the multilayer films, this separation is naturally present due to the additive nature of the imperfections. As shown in Figure S8, gradient flow is achieved through the deterministic node (ideal layer parameters). Thus, the stochastic node (random imperfections) does not create to a bottleneck for backpropagation. The additive nature of random errors enables backpropagation of the gradients in the optimization process.


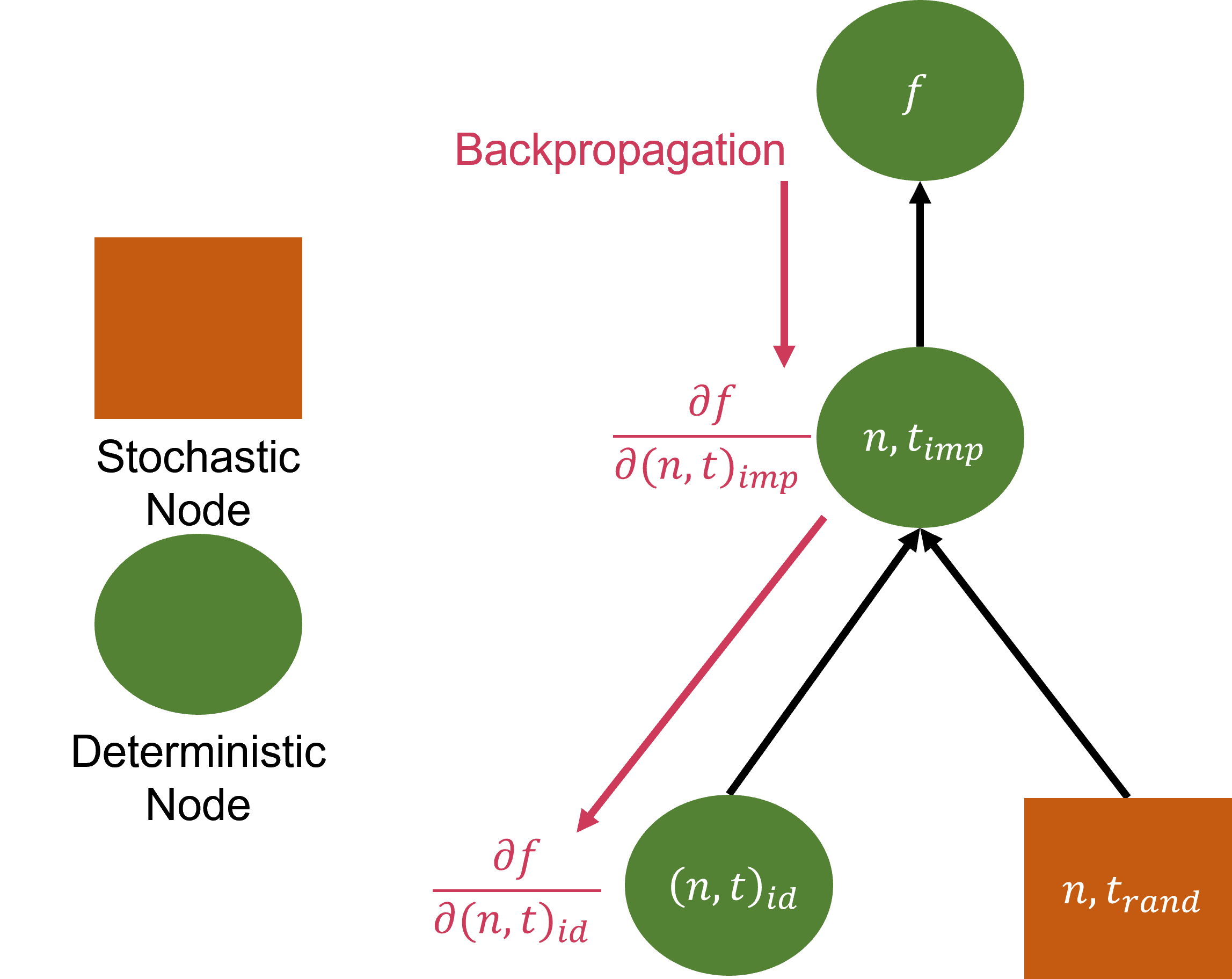


Figure S8. Backpropagation of gradients with random fabrication imperfections integrated to the NN-based multilayer film optimization.

1. References

[1] D. P. Kingma and J. L. Ba, “Adam: A Method for Stochastic Optimization,” *3rd International Conference on Learning Representations, ICLR 2015 - Conference Track Proceedings*, Dec. 2014, doi: 10.48550/arxiv.1412.6980.

[2] C. Liu, L. Zhu, and M. Belkin, “Loss landscapes and optimization in over-parameterized non-linear systems and neural networks,” *Appl Comput Harmon Anal*, vol. 59, pp. 85–116, Feb. 2020, doi: 10.48550/arxiv.2003.00307.

[3] S. Arora, N. Cohen, and E. Hazan, “On the Optimization of Deep Networks: Implicit Acceleration by Overparameterization.” PMLR, pp. 244–253, Jul. 03, 2018. Accessed: Nov. 30, 2022. [Online]. Available: https://proceedings.mlr.press/v80/arora18a.html

[4] I. Safran, G. Yehudai, and O. Shamir, “The Effects of Mild Over-parameterization on the Optimization Landscape of Shallow ReLU Neural Networks,” Jun. 2020, doi: 10.48550/arxiv.2006.01005.

[5] Y. Li, T. Ma, and H. Zhang, “Algorithmic Regularization in Over-parameterized Matrix Sensing and Neural Networks with Quadratic Activations,” Dec. 2017, doi: 10.48550/arxiv.1712.09203.

[6] Z. Allen-Zhu, Y. Li, and Z. Song, “A Convergence Theory for Deep Learning via Over-Parameterization,” *36th International Conference on Machine Learning, ICML 2019*, vol. 2019-June, pp. 362–372, Nov. 2018, doi: 10.48550/arxiv.1811.03962.

[7] S. Arora, S. S. Du, W. Hu, Z. Li, and R. Wang, “Fine-Grained Analysis of Optimization and Generalization for Overparameterized Two-Layer Neural Networks.” PMLR, pp. 322–332, May 24, 2019. Accessed: Nov. 30, 2022. [Online]. Available: https://proceedings.mlr.press/v97/arora19a.html

[8] D. P. Kingma and M. Welling, “Auto-Encoding Variational Bayes,” *2nd International Conference on Learning Representations, ICLR 2014 - Conference Track Proceedings*, Dec. 2013, doi: 10.48550/arxiv.1312.6114.
